# Caffeic Acid Phenyl Ester (CAPE) Protects against Iron-Mediated Cellular DNA Damage through Its Strong Iron-Binding Ability and High Lipophilicity

**DOI:** 10.3390/antiox10050798

**Published:** 2021-05-18

**Authors:** Bo Shao, Li Mao, Miao Tang, Zhu-Ying Yan, Jie Shao, Chun-Hua Huang, Zhi-Guo Sheng, Ben-Zhan Zhu

**Affiliations:** 1 Department of Public Health, Jining Medical University, Jining 272067, China; shaobo@mail.jnmc.edu.cn; 2 State Key Laboratory of Environmental Chemistry and Ecotoxicology, Research Center for Eco-Environmental Sciences, The Chinese Academy of Sciences, Beijing 100085, China; miaotang_st@rcees.ac.cn (M.T.); zyyan_st@rcees.ac.cn (Z.-Y.Y.); jieshao@rcees.ac.cn (J.S.); chhuang@rcees.ac.cn (C.-H.H.); zgsheng@rcees.ac.cn (Z.-G.S.); 3 University of Chinese Academy of Sciences, Beijing 100085, China; 4 Joint Institute for Environmental Science, Research Center for Eco-Environmental Sciences and Hong Kong Baptist University, Beijing 100085/Hong Kong 999077, China

**Keywords:** caffeic acid phenethyl ester, iron, DNA damage, hydroxyl radical, redox-inactive iron complex, lipophilicity

## Abstract

Caffeic acid phenethyl ester (CAPE) and its structurally-related caffeic acid (CA), ferulic acid (FA) and ethyl ferulate (EF) are constituents of honeybee propolis that have important pharmacological activities. This study found that CAPE—but not CA, FA, and EF—could effectively prevent cellular DNA damage induced by overloaded iron through decreasing the labile iron pool (LIP) levels in HeLa cells. Interestingly, CAPE was found to be more effective than CA in protecting against plasmid DNA damage induced by Fe(II)–H_2_O_2_ or Fe(III)–citrate–ascorbate-H_2_O_2_ via the inhibition of hydroxyl radical (•OH) production. We further provided more direct and unequivocal experimental evidences for the formation of inactive CAPE/CA–iron complexes. CAPE was found to have a stronger iron-binding ability and a much higher lipophilicity than CA. Taken together, we propose that the esterification of the carboxylic moiety with phenethyl significantly enhanced the iron-binding ability and lipophilicity of CAPE, which is also responsible for its potent protection against iron-mediated cellular DNA damage. A study on the iron coordination mechanism of such natural polyphenol antioxidants will help to design more effective antioxidants for the treatment and prevention of diseases caused by metal-induced oxidative stress, as well as help to understand the structure–activity relationships of these compounds.

## 1. Introduction

Iron is essential for life due to the efficiency with which it converts between its Fe(II) and Fe(III) redox states. Iron participates in oxygen transport, DNA synthesis, electron transport, and many other biological processes. However, iron overload could cause several adverse effects in many biological systems [1,2,3]. In humans, an increase of cellular-free iron has close relationships with stroke, aging, Alzheimer’s, Parkinson’s, and cardiovascular diseases [4,5,6]. Highly reactive •OH formation via Fenton reactions is critical for excessive iron-mediated cell damage [7,8,9,10,11,12,13]. •OH could induce protein, lipid, and DNA damage, which comprise one of the major sources of oxidative stress [14,15]. Iron-induced DNA damage through •OH is closely related to the cellular death and tissue damage caused by several diseases as mentioned above. Thus, the inhibition of iron-induced DNA damage by •OH has important biological significance for the prevention of diseases.

Polyphenol compounds widely found in tea, fruits, and vegetables have been considered to be good antioxidants [16,17,18]. Both the scavenging of ROS and metal binding by polyphenols are major mechanisms of their antioxidant activity, especially the metal-binding ability [19,20,21,22,23,24]. Tannic acid was found to prevent the Fenton reaction by chelating Fe^2+^ [25]. Polyphenols also could prevent nuclear DNA damage induced by iron through the metal-binding ability, and catechol derivatives have exhibited more antioxidant properties than those without the catechol moiety [26].

Hydroxycinnamic acid is an important subgroup of phenolic compounds that is widely found in plants and plant products [27]. They have significant antioxidant activity both in vitro and in vivo [27,28]. Recently, caffeic acid phenethyl ester (CAPE), which was extracted from propolis, was found to have interesting pharmacological activity. CAPE may play an important role in the chemo-preventive and anti-tumor properties of propolis [29,30]. CAPE has also been shown to prevent lipid peroxidation in the spinal cord and brain [31,32]. An extract of propolis with CAPE was found to have more potent antioxidant activity than an extract without CAPE [33]. Though attempts have been made to investigate the mechanism of the antioxidant property of CAPE, few detailed studies have ever been done on its metal-chelating characteristic and protection against iron-mediated cellular DNA damage via •OH production. Because of the catechol-moiety of CAPE, we speculate that metal-chelating character may play a key role in its antioxidant activity. 

**Figure 1:**
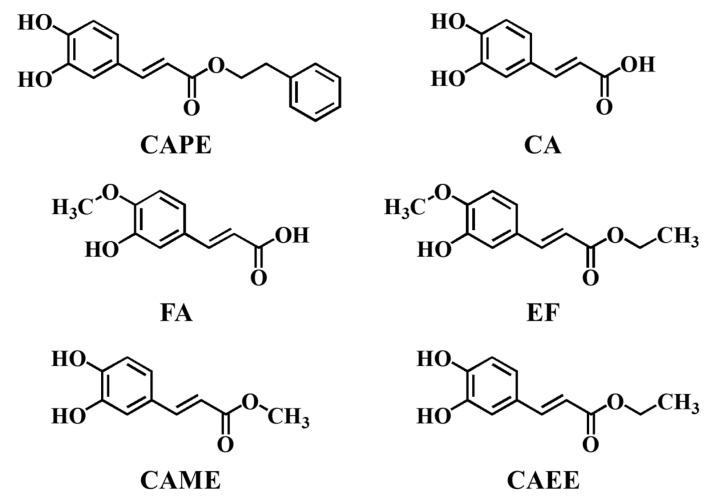
Structural formulas of CAPE, CA, FA, EF, CAME, and CAEE.

Therefore, this study was designed to address the following questions: Can CAPE and its analogues including CA, FA, and EF prevent iron-mediated DNA damage from •OH in cellular DNA (Figure 1), and if so, what is the underlying molecular mechanism?

## 2. Materials and Methods 

### 2.1. Materials 

CAPE (97%), CA (98%), FA (98%), EF (98%), caffeic acid methyl ester (CAME) (98%), caffeic acid ethyl ester (CAEE) (98%), calf thymus DNA (ct-DNA), ascorbic acid, and bovine serum albumin (BSA) were purchased from Sigma (St. Louis, MO, USA). Ferric chloride (≥99%), ferrous ammonium sulfate hexahydrate (≥99.5%), and trisodium citrate dihydrate (≥99.5%) were purchased from Sinopharm Chemical Reagent Co., Ltd. Calcein and calcein-AM were purchased from MedChemExpress (MCE). 5,5-dimethyl-1-pyrroline N-oxide (DMPO) was purchased from DOJINDO Molecular Technologies. 

### 2.2. Immunofluorescence Analysis of Intracellular γ-H2AX Generation in HeLa Cells 

The immortalized human cervical carcinoma HeLa cell line used in this study was obtained from the Cell Resource Center, Peking Union Medical College. The cell line was checked to be free of mycoplasma contamination by PCR and culture. Its species origin was confirmed with PCR. The identity of the cell line was authenticated with STR profiling (FBI, CODIS). HeLa cells were cultured in a DMEM medium (4 mM L-glutamine, 4500 mg/L glucose, and no sodium pyruvate) with 10% fetal bovine serum (FBS) and 1% penicillin–streptomycin at 37 °C under a 5% CO_2_ atmosphere. 

HeLa cells (10^4^/well) were loaded by incubation with 20 μM Fe(II) for 2 h at 37 °C. Subsequently, cells were incubated with or without 20 μM CAPE and its analogues for 30 min, and then they were incubated with 100 μM H_2_O_2_ for 30 min before fixation by 100% ice-cold methanol for 5 min. For a detailed experimental procedure, please refer to the Supplementary Materials and our previous report [34]. The experiment was repeated three times. The observation of the fluorescence of γ-H2AX in HeLa cells was conducted with a confocal laser scanning microscope (CLSM) (Leica TCS SP). Thirty cells in each group were randomly selected in different microscopic fields for quantitative detection. In detail, each well was equally divided into nine fields and numbered sequentially from the top right corner. Four fields were randomly selected according to a random number table. Thirty cells were then randomly selected from these four fields. All images were analyzed blind regarding exposure parameters, which were coded by one person and read by another who was blinded to the code. The fluorescence intensity of the γ-H2AX signal on selected images was automatically analyzed using the Image-J software. The Statistical Product and Service Solutions (SPSS 21) program was employed to analyze the data through ANOVA. Significant difference from iron-overloaded group are marked with * *p* < 0.05, ** *p* < 0.01, and *** *p* < 0.001.

### 2.3. Labile Iron Pool (LIP) Determination 

HeLa cells (10^6^/mL) were loaded by incubation with 20 μM Fe(II) for 2 h at 37 °C. Subsequently, cells were collected by centrifugation (1000 rpm for 5 min) and incubated with 0.5 μM calcein-AM for 15 min at 37 °C in a D’hanks buffer of pH 7.4. Cells were then washed twice by D’hanks buffer (pH 7.4) and incubated with or without CAPE and its analogues. The fluorescence was monitored by both flow cytometry (BD FACS Caliber) and a CLSM (Leica TCS SP). The experiment was repeated three times.

### 2.4. DNA Strand Breakage 

Plasmid DNA agarose gel electrophoresis [35] was employed to investigate protective effect of CAPE and its analogues against DNA strand breakage induced by an iron-mediated system. For experimental procedure details, please refer to the Supplementary Materials.

### 2.5. Electron Spin Resonance (ESR) Spin-Trapping Studies

The basic system used in this study consisted of Fe(III)/Fe(II), ascorbate, H_2_O_2_, and the spin trapping agent DMPO (100 mM) in 20 mM HEPES (pH 7.4) with or without CAPE and its analogues. For typical parameters, please refer to the Supplementary Materials.

### 2.6. UV–Visible Spectra Measurement 

The reaction mixture involved different concentrations of CAPE and Fe(III)–citrate in 20 mM HEPES (pH 7.4). For the detailed experimental procedure, please refer to our previous report [35].

### 2.7. Calcein Fluorescence Detection

In the presence of calcein, mixtures of CAPE and Fe(III)/Fe(II) with different concentrations in 20 mM HEPES (pH 7.4) were used for fluorescence detection (Ex: 488 nm; Em: 515 nm). Fluorescence spectra were recorded using a Cary Eclipse fluorescence spectrophotometer (Varian, Palo Alto, CA, USA).

### 2.8. Fourier Transform Ion Cyclotron Resonance Mass (FT-ICR-MS) Detection 

The formation of CAPE/CA–Fe(III) complexes was detected by FT-ICR-MS (Bruker Daltonik, GmbH, Bremen, Germany). For the detailed experimental procedure, please refer to the Supplementary Materials and our previous study [35].

### 2.9. Measurement of the Fe(III)-Binding Affinity with Ligands Including CAPE, CA, CAME, and CAEE

The binding affinity of Fe(III) with ligands (K_b(Fe(III)-ligands)_) was studied with a fluorescence displacement method using calcein as the fluorescent agent. As we know, calcein can be used as a fluorescence probe due to the fluorescence quenching upon interaction with iron. When ligands compete to bind iron from calcein, the fluorescence quenching of calcein–iron is inhibited. Thus, calcein was used as fluorescent probe here to measure the iron-binding affinity. In the solution (100 mM PB at pH7.4), Fe(III) and calcein were kept at 0.5 and 20 μM, respectively. IC_50_ represents the 50% inhibitory concentration of each ligand. The binding constants between each ligand and Fe(III) were calculated with the following equation [36,37]:

*K*_d_ = [*IC*_50_]/(1 + [probe]/*K*_Probe(Calcein)_) (1)

(2) $$K_{b(\mathrm{Fe}(\mathrm{III})-\mathrm{Ligand})}=\frac{1}{Kd}$$

where [probe] is the concentration of calcein (0.5 μM) and the intrinsic calcein-binding constant *K*_b(Fe(III)-Calcein)_ of Fe(III) is 10^24^ M^−1^. *K*_probe(Calcein)_ is the dissociation constant for the intercalation of Fe(III) with calcein, so *K*_probe(Calcein)_ = 1/10^24^ M^−1^.

### 2.10. Cyclic Voltammetry (CV) Measurement 

CV was performed in 100 mM PB (pH 7.4) on a Princeton Applied Research (VersaSTAT3) potentiostat with a Pt wire as the counter electrode, a saturated calomel electrode (SCE) as the reference electrode, and a glassy carbon electrode (5 mm in diameter) as the working electrode. The scan rate was 2 mV/s.

### 2.11. Partitioning Study

Organic solvents (1-octanol)/aqueous (100 mM phosphate buffer at pH 7.4) phase partition for CAPE and its analogues were conducted using the shake-flask method [38], with the concentration in each phase determined by a Cary 3500 UV–Vis spectrophotometer (Agilent, Santa Clara, CA, USA).

### 2.12. Statistical Analysis

All experiments were repeated three times. For the figures showing immunofluorescence staining and LIP quantitative analysis, the data were obtained from three parallel experiments. The fluorescence intensities were divided by control group, and the fold values compared to control were analyzed by the SPSS 21 program. ANOVA followed by a least significant difference (LSD) test (*p* < 0.05, 0.01, or 0.001) was employed to determine significant differences between different treatments. Significant differences from Fe(II)–H_2_O_2_ group were marked with * *p* < 0.05, ** *p* < 0.01, or *** *p* < 0.001. For the data showing ESR spectra, MS spectra, fluorescence spectra, and UV–Vis absorption spectra, the experiments were repeated three times, and only typical spectra are presented.

## 3. Results and Discussion

### 3.1. CAPE Was Found to Be the Most Potent in Protecting Against Iron-Mediated Cellular DNA Damage

**Figure 2:**
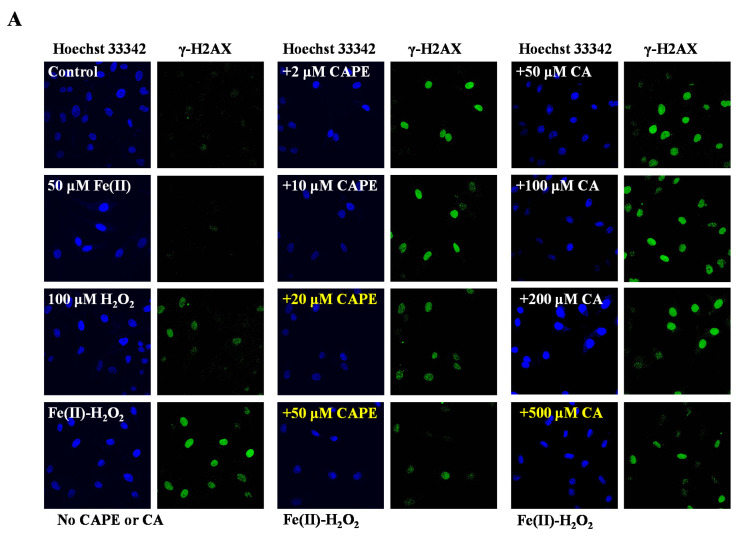

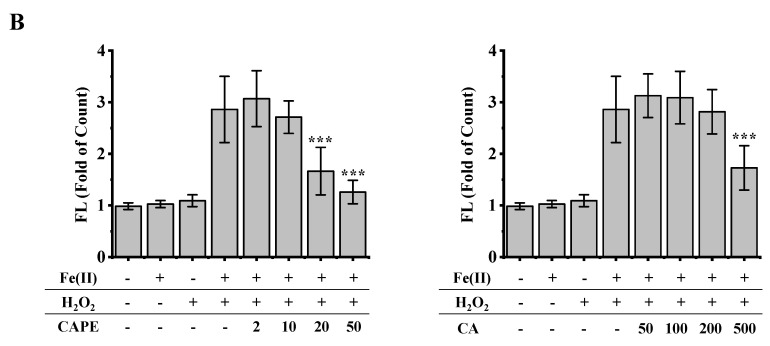
Inhibition of CAPE and its analogues on the formation of γ-H2AX induced by iron-overload in HeLa cells, as measured by immunofluorescence staining. (**A**) Representative images of HeLa cells stained for Hoechst 33342 (nuclear stain) monoclonal antibodies of γ-H2AX under different conditions as indicated. (**B**) Quantitation of γ-H2AX formation in HeLa cells under different conditions as indicated. *** Significant difference from the Fe(II)–H_2_O_2_ groups, *p* < 0.001. The experiment was repeated three times, and thirty cells were randomly selected in different microscopic fields for statistical analysis in each group.

It is well-known that iron-induced DNA damage through •OH is closely related to oxidative stress and cellular death, which might induce tissue damage and several diseases [1,4,5,6]. We first investigated whether CAPE and its analogues could inhibit cellular DNA damage induced by an iron-overload system in HeLa cells [39]. As shown in Figure 2, neither iron overload nor H_2_O_2_ alone could induce significant cellular DNA damage, while double strands breaks (DSBs) were obviously produced in iron-overloaded cells in the presence of H_2_O_2_. Interestingly, the pre-treatment of 2–50 μM CAPE could significantly inhibit the cellular DNA damage induced by iron overload (with H_2_O_2_) in a concentration-dependent manner. The results observed from Fe(II)–H_2_O_2_–20 μM CAPE and Fe(II)–H_2_O_2_–50 μM CAPE exhibited significant differences with the Fe(II)–H_2_O_2_ group. In contrast, for CA (one of CAPE analogues), only 500 μM CA could provide similar protection against DNA damage induced by iron overload (with H_2_O_2_). However, for another two CAPE analogues (FA and EF), no significant inhibition could be observed (Figure S1).

### 3.2. CAPE Was Found to Be the Most Potent in Decreasing LIP Levels Induced by Iron Overload 

Previous reports suggested that the catechol group might play a critical role in the metal-binding ability. We speculated that the inhibition of CAPE on the oxidatively generated DNA damage might have been due to its ability to chelate overloaded iron and inhibit its redox activity. Therefore, we further studied the level of LIP in iron-overloaded HeLa cells pre-treated with or without CAPE and its analogues. 

LIP was first proposed as a transition pool between extracellular iron and protein-related cellular iron [40], which can participate in the redox cycle but can be eliminated by the chelating agent. Though the level of LIP can change dynamically with stimulation, the changes are temporary and homeostatic. However, in the case of iron overload or deprivation, these changes may disrupt cell homeostasis and compromise its integrity [41].

**Figure 3:**
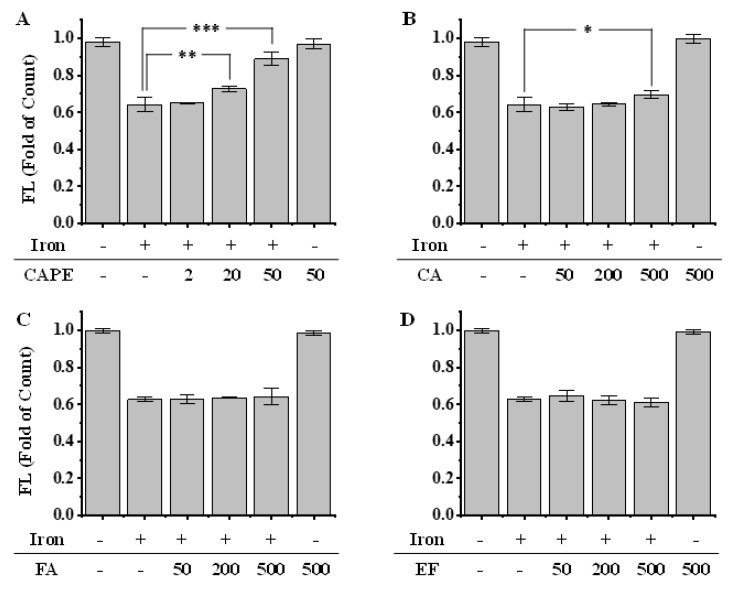
Effect of CAPE and its analogues on the LIP level in iron-overloaded cells, as measured by flow cytometry (**A**–**D**). HeLa cells were pretreated with 20 μM Fe(II) for 2 h and then incubated with calcein-AM. After 15 min, cells were washed and incubated with or without CAPE and its analogues for 15 min. The fluorescence intensity of cells was measured by flow cytometry. Significant differences from the iron-overloaded group are marked with * *p* < 0.05, ** *p* < 0.01, and *** *p* < 0.001. The experiment was repeated three times.

As shown by both confocal laser scanning microscopy and flow-cytometry, the pre-treatment of Fe(II) for 2h significantly decreased the fluorescence intensity of calcein, which indicated the increase of LIP in HeLa cells (Figure 3 and Figure S2). However, the fluorescence significantly regenerated after the addition of 20–50 μM CAPE (*p* < 0.001), which suggested that a low concentration of CAPE could decrease the LIP levels, possibly by chelating with LIP (Figure 3). In contrast, a much higher concentration of CA (500 μM) was required to increase the fluorescence (*p* < 0.05), while no effect was observed with EF and FA (50–500 μM) (Figure 3B–D).

Based on the results, we suggested that the inhibition of CAPE on oxidatively generated DNA damage was likely due to its ability to chelate overloaded iron and inhibit its redox activity. The catechol group of CAPE may play an important role in its iron-chelating ability. However, CA, which also has catechol group, exhibited a much weaker protective effect on iron-overloaded cellular DNA damage. This not only suggested that the protective effect depends on the ortho-dihydroxy group but also that the substituents at the para position of phenolic hydroxyl may play a critical role. To test the above hypothesis, we also employed two CAPE analogues, CAME and CAEE, to investigate their protective effect against iron overload-induced cellular DNA damage (Figure 1). As shown in Figure S3, 100–500 μM CAME or 50–500 μM CAEE could significantly inhibit iron overload-induced DNA damage, and their protective effect was found to be stronger than CA but weaker than CAPE. Similarly, we also found that 200–500 μM CAME or 50–500 μM CAEE could significantly regenerate the fluorescence of calcein (Figure S4), suggesting that CAME and CAEE (higher concentration) could also inhibit iron overload-induced DNA damage by decreasing LIP levels. Interestingly, although CAPE, CA, CAME, and CAEE have an ortho-dihydroxy group on their benzene rings, their protective effects were markedly different. These results confirmed that the protective effect may depend on the ortho-dihydroxy group, and the substituents at the para position of phenolic hydroxyl could also play a critical role. Based on the aforementioned considerations, we further investigated the protection of CAPE and its analogues against iron-mediated oxidative damage on isolated and purified DNA. 

### 3.3. CAPE Was Found to Be More Effective Than CA in Protecting Against Iron-Mediated DNA Damage as Measured by DNA Strand Breaks

**Figure 4:**
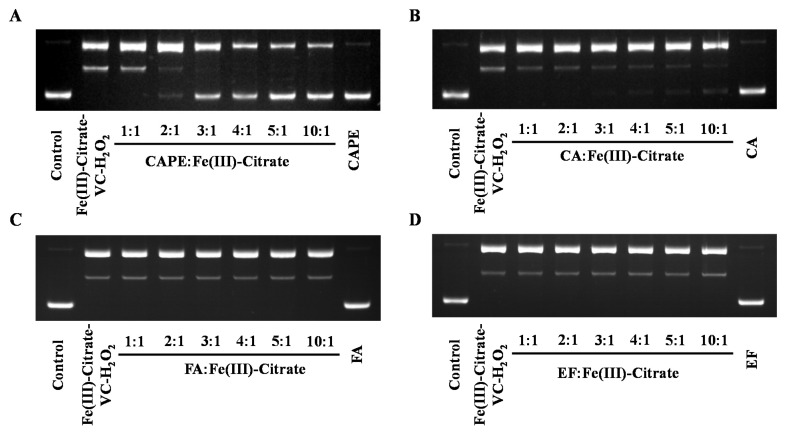
Protective effect of CAPE and its analogues against Fe(III)–citrate–ascorbate–H_2_O_2_-induced DNA damage. (**A**) 40–200 μM CAPE significantly inhibited Fe(III)-citrate-Vc-H_2_O_2_ induced DNA damage. (**B**) 20–200 μM CA slightly inhibited Fe(III)-citrate-Vc-H_2_O_2_ induced DNA damage. (**C**,**D**) FA and EF exhibited no protective effect on Fe(III)-citrate-Vc-H_2_O_2_ induced DNA damage. The reaction mixture contained 20 μM Fe(III)–citrate, 10 μM ascorbate, and 200 μM H_2_O_2_, as well as the indicated concentrations of CAPE and its analogues. The experiments were repeated three times, and only typical images are presented.

Plasmid DNA (pBR322) was employed to study whether CAPE and its analogues could protect against iron-mediated DNA strand breakage by •OH. Because of the facile hydrolysis of Fe(III) in an aqueous and neutral solution, an Fe(III)–citrate complex was used to study the protective effect of CAPE against DNA damage. Citrate anions are thought to be abundant in biological fluids, and Fe(III)–citrate has been considered as one of the major components of LIPs in cells [42,43]. As observed in Figure 4A, upon the addition of Fe(III)–citrate/ascorbate/H_2_O_2_, the supercoiled DNA decreased and converted into the relaxed circular and linear DNA. However, in the presence of CAPE, Fe(III)–citrate/ascorbate/H_2_O_2_-induced DNA strand breaks decreased significantly in a molar ratio (CAPE:Fe(III))-dependent manner. We found that DNA cleavage decreased with increasing ratios of CAPE:Fe(III); when the ratio increased over 2:1, the linear form of plasmid DNA completely disappeared and the supercoiled form markedly increased. Meanwhile, the protective effect of CA in this system was found to be lower than CAPE, while no significant protective effect was observed for FA or EF. As shown in Figure S5, CAPE also significantly inhibited Fe(II)–H_2_O_2_-induced DNA strand breaks depending on the ratio. The protective activity of CAPE and its analogues decreased in the following order: CAPE > CA > FA ≈ EF. This was similar to the trend of their protective activity against Fe(III)–citrate/ascorbate/H_2_O_2_-induced DNA damage. 

These results indicated that CAPE and CA exhibit different protective effects against iron (Fe(II)/Fe(III))-mediated oxidatively generated DNA damage, and the protection of CAPE is stronger than CA. FA and EF exhibited no protective effect against iron-mediated DNA damage.

**Figure 5:**
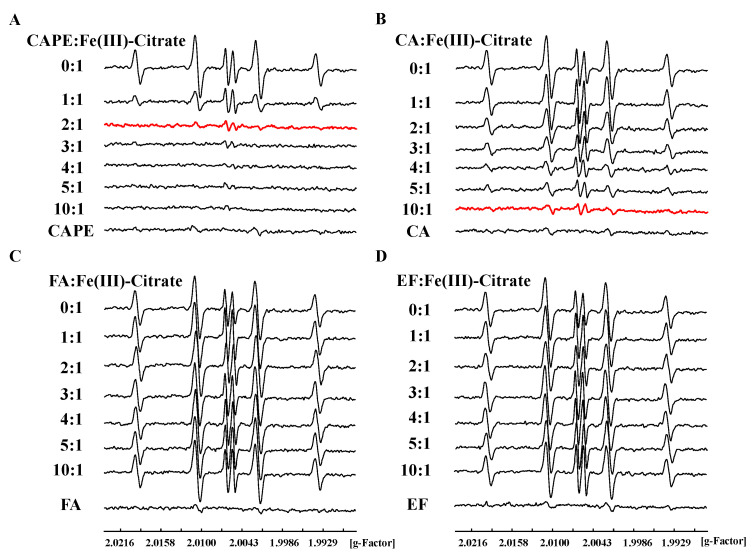
Inhibitory effect of CAPE and its analogues against Fe(III)–citrate–Vc–H_2_O_2_-induced •OH generation. (**A**): 200 μM CAPE completely inhibited Fe(III)-citrate-Vc-H_2_O_2_ induced •OH generation. (**B**): 300-1000 μM CA significantly inhibited Fe(III)-citrate-Vc-H_2_O_2_ induced •OH generation. (**C**,**D**): FA and EF exhibited no inhibitory effect against Fe(III)-citrate-Vc-H_2_O_2_ induced •OH generation. The reaction mixture contained 100 μM Fe(III)–citrate, 100 μM ascorbate, and 1 mM H_2_O_2_, as well as the indicated concentrations of CAPE and its analogues. The experiments were repeated three times, and only typical spectra are presented.

The ESR spin trapping method was also used to study the inhibition of CAPE and its analogues on the production of •OH in an iron-mediated system. We found that DMPO/•OH and DMPO/•CH_3_ adducts could be produced by Fe(III)–citrate–ascorbate–H_2_O_2_ in the presence of the spin-trapping agent DMPO and •OH scavenger dimethyl sulfoxide (DMSO) (Figure S6), and the central part of the spectrum in Fe(III)–citrate–ascorbate system is ascorbyl radical (asc^•−^) [44]. As shown in Figure 5, DMPO/•OH formation induced by an Fe(III) or Fe(II) system significantly decreased in the presence of CAPE or CA, though not with EF and FA. The inhibition of •OH production by CAPE/CA was found to depend on the molar ratio of CAPE (CA):Fe(III)/(Fe(II)). The production of •OH decreased with the increasing of the ratios. When the molar ratio of CAPE to Fe(III) increased to 2:1 or CA to Fe(III) increased to 10:1, •OH production induced by Fe(III)–citrate/ascorbate/H_2_O_2_ was completely inhibited (Figure 5). In an Fe(II)/H_2_O_2_ system, CAPE also could completely inhibit •OH production when the ratio of CAPE to Fe(II) increased to 3:1; however, even when the ratio of CA:Fe(II) increased to 20:1, significant •OH production was still observed (Figure S8). 

The above results indicated that CAPE and CA, but not EF and FA, could inhibit iron-mediated •OH production in a ratio-dependent manner, which is similar with DNA damage inhibition results in vitro. We also found that CAME and CAEE could protect against iron-mediated DNA damage through inhibiting •OH generation, but their protective effect was weaker than CAPE (Figures S9–S12). Therefore, we concluded that the ortho-dihydroxy group on the benzene rings of CAPE and CA plays an important role in iron-mediated DNA damage in vitro, indicating that they may chelate iron and affect its redox activity. 

However, the direct and systematic experimental evidence on the binding effect of CAPE/CA with iron is very limited so far. It is currently uncertain whether CAPE/CA–iron complexes are formed; if so, what are the molar ratios of CAPE/CA–iron and their redox activity compared to iron alone? On the other hand, we found that the protective effect of CAPE and its analogues against DNA damage induced by an iron-containing system in vitro was different from that in HeLa cells: both CAPE and CA could protect against iron-mediated oxidative damage on isolated and purified DNA (plasmid DNA and ct-DNA), while only CAPE could markedly inhibit the cellular DNA damage induced by iron overload. The LIP analysis also indicated that only CAPE could diminish the fluorescence quenching induced by overloaded iron. Since both CAPE and CA have a catechol group and the ability to chelate iron, why did they show different protective effects in cellular DNA? In order to elucidate these questions, we further investigated the iron-chelating ability of CAPE and its analogues via different analytical methods such as UV–Vis, fluorescence, oxygen consumption, and FT-ICR-MS analysis.

### 3.4. CAPE Was Found to Have Strong Iron-Binding Ability, Leading to the Formation of Redox-Inactive Iron–CAPE Complex 

**Figure 6:**
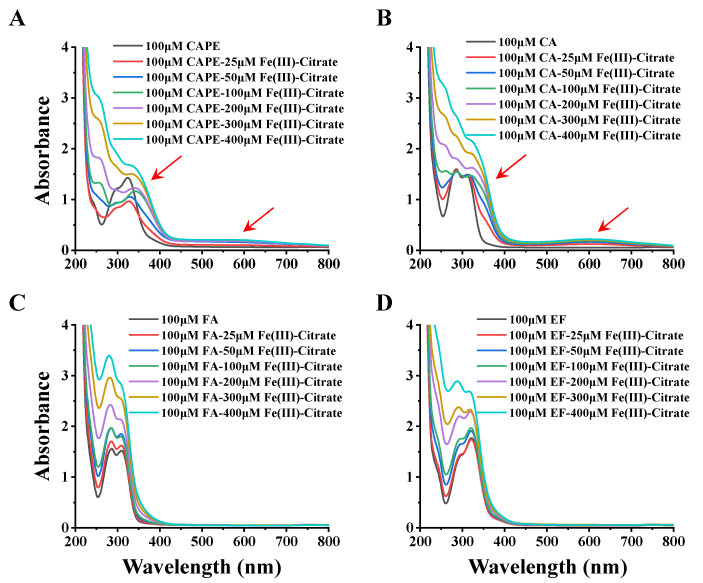
UV spectral changes observed upon the addition of Fe(III)–citrate to CAPE and its analogues. (**A**) the incremental addition of Fe(III)–citrate (25–400 μM) to 100 μM CAPE decreased the absorbance peak at 322 nm, with concomitant increases at 350 and 570 nm. (**B**) the incremental addition of Fe(III)–citrate (25–400 μM) to 100 μM CAPE generated new absorbance peaks at 372 and 610 nm. (**C**,**D**) No significant new absorbance peak was observed in FA and EF with Fe(III)–citrate. The reaction mixture contained 100 μM CAPE or its analogues and different concentrations of Fe(III)–citrate (25–400 μM). The experiments were repeated three times, and only typical spectra are presented.

In Figure 6, the incremental addition of Fe(III)–citrate (25–400 μM) to 100 μM CAPE gradually decreased the absorbance peak at 322 nm, with concomitant increases at 350 and 570 nm. Meanwhile, the UV–visible absorption spectra of CA and Fe(III) were similar to that of CAPE: after the addition of Fe(III)–citrate, new absorbance peaks at 372 and 610 nm appeared. These spectra changes suggested that both CAPE and CA could form CAPE/CA–iron complexes with iron, which have different characteristic absorption peaks than CAPE/CA alone. No significant new absorbance peak was observed in FA and EF with Fe(III)–citrate, and the increase of the peaks of FA and EF could be attributed to the control group of Fe(III)–citrate alone, as shown in Figure S13. These spectral changes strongly suggested that the ortho phenolic hydroxy groups of CAPE or CA are essential for their iron coordination. 

The interactions of CAPE and its analogues with Fe(III)–citrate were further determined by the fluorescence method. The principle of the test is based on the binding of calcein to iron in a stoichiometric manner, which results in a fluorescence quenching. Therefore, a lower reading in fluorescence intensity indicates a higher level of iron in biological fluids [45,46]. The affinity constants for calcein are 10^14^ and 10^24^ M^−1^ for Fe(II) and Fe(III), respectively [47]. Furthermore, calcein acetoxymethyl ester (calcein-AM) can also be used to estimate LIP in cells due to the high cell membrane permeability of calcein-AM [48,49,50]. 

**Figure 7:**
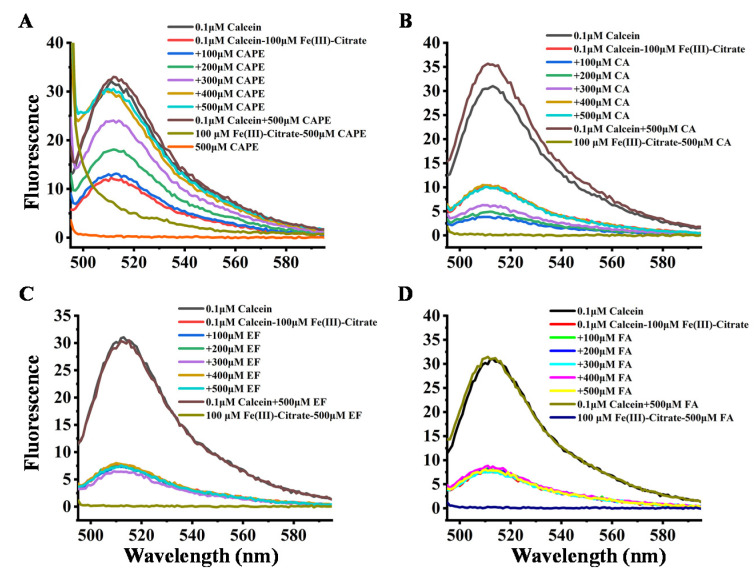
Effect of CAPE and its analogues on the fluorescence intensity of calcein–Fe(III)–citrate. (**A**,**B**) the fluorescence quenching of calcein by Fe(III)–citrate diminished in a (CAPE/CA:Fe(III)) ratio-dependent manner. (**C**,**D**) EF and FA exhibited no inhibition of fluorescence quenching by Fe(III)–citrate. The reaction mixture contained 0.1 μM calcein, 100 μM Fe(III)–citrate, and indicated concentrations of CAPE or its analogues (100–500 μM). The experiments were repeated three times, and only typical spectra are presented.

The quenching effect of iron on calcein fluorescence was examined by measuring the changes in the fluorescence intensity of calcein after mixing iron (100 μM) with calcein (1 μM) in a 20 mM HEPES buffer at pH 7.4. As shown in Figure 7, the fluorescence of 0.1 μM calcein was significantly quenched by 100 μM Fe(III)–citrate, which could be attributed to the binding between calcein and Fe(III). However, after the addition of CAPE, the fluorescence quenching by Fe(III)–citrate gradually diminished in a (CAPE:Fe(III)) ratio-dependent manner, and when the ratio increased to over 3:1, the fluorescence quenching was completely abolished by CAPE. CAPE alone could neither quench nor enhance the calcein fluorescence intensity. However, CA showed a weaker inhibition of fluorescence quenching by Fe(III)–citrate, even when the ratio increased to 5:1. FA and EF showed no inhibitory effects. The analogous inhibitory effect of CAPE and its analogues on the fluorescence quenching by Fe(II) can also be observed in Figure S14: CAPE significantly diminished the fluorescence quenching when the ratio increased to 4:1. The inhibitory effect of CAPE on fluorescence quenching by Fe(III)–citrate or Fe(II) resulted from competitive iron binding with CAPE and calcein. However, CA exhibited a weaker inhibitory effect on fluorescence quenching by iron, which had a similar chemical structure to CAPE. This result indicated that the affinity of CA to iron is weaker than CAPE. 

Dissolved oxygen consumption was also used to investigate the redox activity of CAPE/CA–iron complexes. We found that the redox reaction between ascorbic acid and Fe(III)–citrate induced significant oxygen consumption and that CAPE significantly decreased oxygen consumption as the ratio of CAPE:Fe(III) increased (from approximately 1:1 to 3:1), suggesting that CAPE may bind with Fe(III) to form a redox-inactive (less reactive) CAPE-Fe(III) complex, as compared with Fe(III)-citrate (Figure S15A). It can be seen in Figure S15B that only when the ratio of CA to Fe(III) increased to 5:1 could a decrease of O_2_ consumption be observed. EF and FA had no significant effects on the O_2_ consumption induced by ascorbic acid/Fe(III)–citrate (Figure S15C,D). 

We also detected the change of redox potential of Fe(III) after being chelated by CAPE or CA through cyclic voltammetry methods. Figure S16 shows that Fe(III) alone exhibited its reduction peak at approximately −0.65 V. When Fe(III) was chelated by CAPE, the reduction potential decreased to −1.00 V, indicating that the chelation of CAPE to Fe(III) significantly inhibited the redox activity of Fe(III). Similarly, the chelation of CA, CAME, and CAEE decreased the reduction potential of Fe(III) to −0.76, −0.79, and −0.99 V, respectively, which suggested that all four chelators could inhibit the redox activity of Fe(III) through their chelating effects, as well as that the inhibitory effect of CAPE is stronger than CA. These results were consistent with the optical spectral analyses and dissolved oxygen consumption experiments, which further confirmed that the inhibition of both •OH generation and DNA damage by CAPE or CA in an iron-containing system is due to formation of redox-inactive (or less reactive) iron complexes.

**Figure 8:**
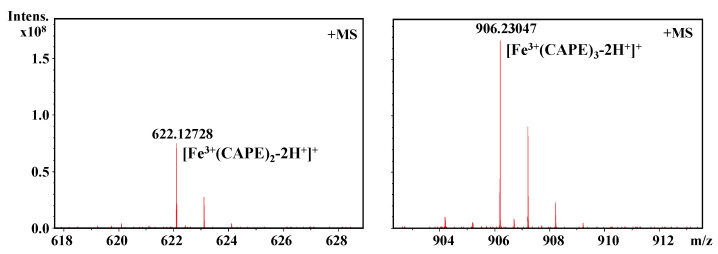
Formation of a CAPE–Fe(III) complex as characterized by FT-ICR-MS analysis. The reaction mixture contained 100 μM CAPE and 10 μM Fe(III) in a 20 mM ammonium acetate buffer (pH 7.4).

**Figure 9:**
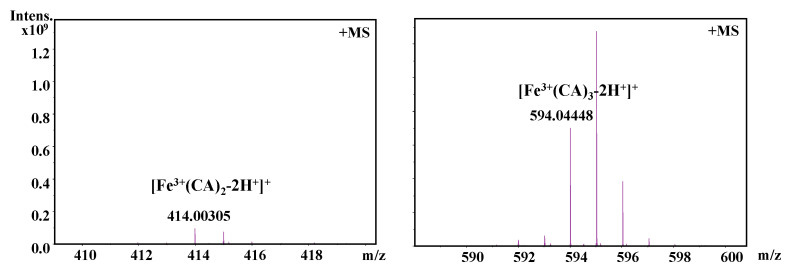
Formation of a CA–Fe(III) complex as characterized by FT-ICR-MS analysis. The reaction mixture contained 100 μM CA and 10 μM Fe(III) in a 20 mM ammonium acetate buffer (pH 7.4).

**Figure 10:**
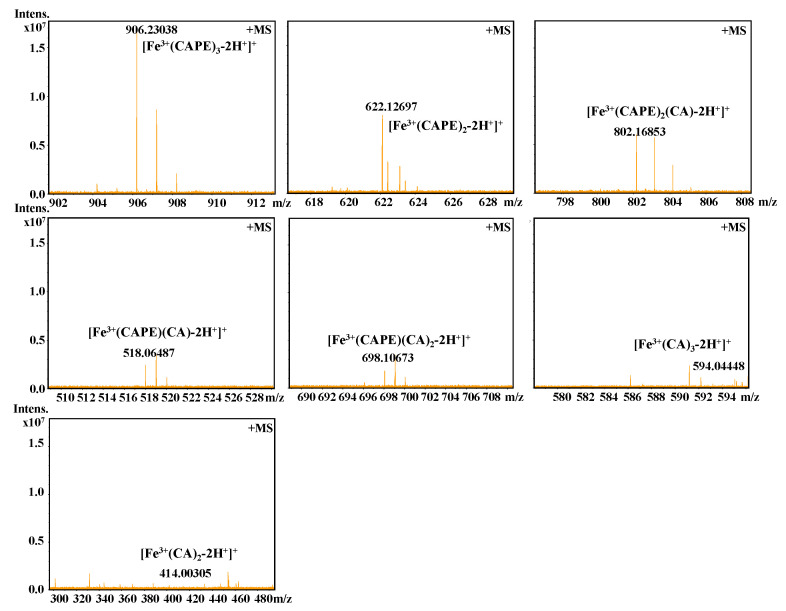
Competitive binding of Fe(III) by CAPE and CA, as characterized by FT-ICR-MS analysis. The reaction mixture contained 100 μM CAPE, 100 μM CA, and 10 μM Fe(III) in a 20 mM ammonium acetate buffer (pH 7.4).

It should be also interesting to know the exact molar ratio of CAPE with Fe(III)/Fe(II). As shown in Figure 8, in the reaction solution involved CAPE and Fe(III), the quasi-molecular ion of the compounds with *m/z* values of 622.12728 and 906.23047, respectively, were detected by FT-ICR-MS in the positive ion mode. Following a comparison of the molecular weights and isotopic distributions, the molecular formula of the compounds with *m/z* at 622.12728 and 906.23047 were assumed to be C_34_H_30_O_8_Fe and C_51_H_46_O_12_Fe, respectively, which could be proposed to be the M^+^ or [M+H]^+^ of the CAPE/Fe(III) complexes formed by 2:1 and 3:1 ratios, respectively. In Figure 9, the molecular formula of the compounds at 414.00305 and 594.04448 were assumed to be C_18_H_14_O_8_Fe and C_27_H_22_O_12_Fe, respectively, which could be assigned as the CA/Fe(III) complexes formed by 2:1 and 3:1 ratios, respectively. These results provided more convincing evidence for the formation of CAPE/CA–iron complexes with molar ratios of 2:1 or 3:1, respectively. Moreover, we measured the competitive binding effect between CAPE and CA with iron. We found that the mixture containing equivalent CAPE and CA preferentially formed CAPE–iron complexes over CA–iron complexes, which also suggested that the iron-cheating ability of CAPE is stronger than CA (Figure 10). 

We further determined their stoichiometry by job’s titration. As shown in Figure S17, the ligands including CAPE, CA, CAME, and CAEE were able to chelate iron at two different molar ratios (2:1 and 3:1 for ligands and iron, respectively). We also investigated their binding constants through titration methods. The binding constants (K_b(iron-ligands)_) between iron and CAPE/CA/CAME/CAEE were studied by the fluorescence displacement method (Table S1; for details, see the Materials and Methods section). In this study, only CAPE was found to be able to effectively compete with calcein to bind iron, while CA/CAME/CAEE exhibited no such competitive effect. The binding constants between each ligand and Fe(III) were calculated with the equation shown in Material and Methods. We found that the K_b(Fe(III)–CAPE)_ was approximately 0.63 × 10^22^ M^−1^, while the K_b_ values of Fe(III) with other three ligands (CA, CAME, and CAEE) were less than 10^20^ M^−1^ (Table S1).

We first systematically studied the interaction of CAPE and its analogues with iron (Fe(III)/Fe(II)), providing more direct and stronger experimental evidence for the formation of inactive (or less reactive) CAPE/CA–Fe(III)/Fe(II) complexes, which have important biological significance for future study on polyphenol-related antioxidant effects.

From the above results, we found that the iron-chelating ability of CAPE is significantly stronger than CA. We speculated that the difference of iron-binding affinity between CAPE and CA may have been due to the following reasons: Compared with carboxylate, phenethyl is a significant electron-donating group [51,52], and the binding constants of metals to ligands increase with the increasing electron-donating ability of the para-substituent [53,54,55]. Therefore, the high iron-binding affinity by CAPE may have a close relationship with the esterification of the carboxylic moiety with phenethyl, which could enhance the electronic density over the oxygen atom and increase its chelating ability. On the other hand, hard–hard or soft–soft combinations are kinetically favored [52]. Within a stronger electron-donor power, CAPE has a lower electron affinity and a higher molecular hardness; accordingly, the Fe(III)-binding constant of CAPE is the largest among the four analogues [52]. Similarly, when the hydrogen atom in the -COOH group was substituted with a methyl or ethyl group, which were also electron-donating groups, its iron-chelating ability was also found to increase [52]. Therefore, the stronger iron-chelating ability of CAPE and redox-inactive CAPE–iron complex formation should be the key factor for its more potent protective effect against iron-overload-mediated cellular DNA damage. 

However, although CA is a weaker chelator with iron, it still could protect iron-mediated DNA damage in purified DNA, which is not consistent with its protective effect in cellular DNA in cells. We speculate that this may be related to the different lipophilicity values of CA and CAPE. 

### 3.5. High Lipophilicity of CAPE Enhanced Its Protective Effect Against Iron-Mediated Cellular DNA Damage

According to previous studies, the lipophilic character of antioxidants is an important factor in their biological activity [56,57,58,59]. Though various polyphenol compounds, which can be found in natural products, have been shown to be excellent antioxidants [60,61,62,63], the lipophilic nature of biological membranes restricts the direct intracellular delivery of many hydrophilic antioxidants that cannot cross the cell membrane and exhibit their antioxidant activities inside a cell [64]. Therefore, in this study, besides the difference of the iron-chelating ability between CAPE and CA, the different lipophilicity values of CAPE and CA may have been other important reasons for their protective effect against cellular DNA damage induced by iron, thus allowing these compounds to cross the cell membrane and chelate excessive cellular iron, which may be modulated by their hydrophobic or hydrophilic groups. 

**Scheme 1:**
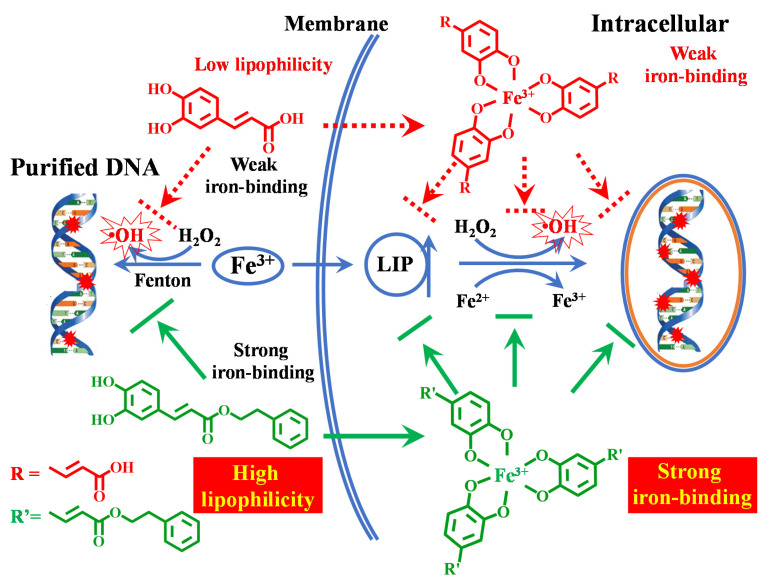
Proposed mechanism of protection by caffeic acid phenyl ester (CAPE) against iron-mediated cellular DNA damage.

As shown in Table 1, the LogK_ow_ value of CAPE (2.01 ± 0.02) was found to be much higher than those of CA (−0.98 ± 0.02), CAME (1.27 ± 0.06), and CAEE (1.51 ± 0.03) (Table 1), which suggested that the esterification of the carboxylic moiety with phenethyl significantly decreases polarity and strongly increased lipophilicity. The lipophilicity of CAPE could facilitate its transport across the cell membrane, where it could exhibit its iron-chelating ability inside the cell [65]. Compared with CAPE, we also found that CA, CAME, and CAEE showed weaker effects on the LIP levels and cellular DNA damage induced by iron. Therefore, although all of them have ortho phenolic hydroxy groups, their lipophilicities are different. Only CAPE could be able to effectively cross the cell membrane and protect against iron-mediated cellular DNA damage through its iron-chelating ability, which can be attributed to the phenethyl esterification of CAPE and its highest lipophilicity among them. Interestingly, since both CAPE and CA have auto-fluorescence (Ex: ~330 nm; Em: ~450nm), we found that the incubation of CAPE with cells could significantly increase its fluorescence inside the cells. However, no significant increase was observed in the CA group (Figure S18), which confirmed that it is indeed much easier for CAPE to cross the cell membrane than CA. These results provided good evidence supporting the strong protective effect of CAPE, but not CA, against iron overload-induced cellular DNA damage (Scheme 1).

In summary, the protective effect of CAPE against DNA damage induced by iron in cells depends not only on its strong iron-binding activity but also on its high lipophilicity. It is noteworthy that both the ortho phenolic hydroxy groups and the substituent at the para position of phenolic hydroxyl have close relationships with the iron-chelating ability and lipophilicity of the phenolic compounds, which are very critical for their protective effect.

### 3.6. Iron-Chelating Ability of CAPE, but Not Its Radical Scavenging Ability, Is Responsible for Its Protection Against Iron-Mediated DNA Damage

The antioxidant properties of natural antioxidants, especially polyphenol compounds, are generally achieved in two pathways: (1) directly scavenging free radicals and (2) chelating transition metals, especially iron, that may inhibit the formation of free radicals [66,67]. It is generally believed that several functional groups of polyphenol compounds are essential for iron chelation: (a) the presence of ortho-dihydroxy groups, (b) the combination of 5-OH and/or 3-OH with a C4 keto group, and (c) a large number of OH groups [68]. Among them, it has been reported that two hydroxy groups in the ortho-position play key roles in the iron chelation and protection of phenolic compounds [26,69,70]. Therefore, in this study, since polyphenols such as CAPE and CA possess ideal structural features for iron chelation, it is reasonable to speculate that the protective effects exerted by CAPE/CA, but not EF and FA, might be due to their iron-chelating activity. As mentioned above, we showed that CAPE could protect against iron-mediated cellular DNA damage by decreasing LIP levels, which supports the important role of the iron-chelating ability of CAPE. Furthermore, we found that the typical lipophilic iron chelator 1,10-phenanthroline (OP) [26,69,70,71,72], but not its hydrophilic analogue bathophenanthroline disulfonate (BPS) (which exhibited no free radical scavenging activities), is also effective in preventing iron-mediated cellular DNA damage through lowing LIP levels (Figures S19–S21). Previous studies have also suggested that both FA and EF have free radical scavenging activities [65], but they exhibited no protective effect against iron-mediated DNA damage in this study. On the other hand, CAPE may hydrolyze to release alcohol, particularly in cells, which might play a critical role in the protective effect through scavenging free radicals. However, as shown in Figure S1, EF exhibited no protective effect on cellular DNA damage, even if it could hydrolyze to release alcohol. Therefore, the contribution of ester hydrolysis to the protective effect in cells can be ruled out. Taken together, our results suggested that the most prominent activity of CAPE in the protection against iron-mediated DNA damage is its strong ability to chelate iron.

### 3.7. Potential Biological Implications

Iron overload has been shown to cause several adverse effects in many biological systems, such as ischemia reperfusion injury, neurodegenerative diseases, and inflammation [1,4,5,6]. Current studies are seeking exogenous ligands to chelate excess iron and to maintain the oxidative balance of cells and tissues. Chelating agents can play a therapeutic role by restricting iron from participating in free radical generation. At the same time, iron-chelating agents can also be used in the development of anti-tumor drugs by consuming excess iron or causing selective oxidative stress in tumor cells [73,74]. For the purpose of exerting their pharmacological effects, the chelating agents must not only have a strong iron affinity but also be able to cross biological membranes to reach the target site and exert their pharmacological effects [75]. 

In this study, both the strong iron-binding ability and high lipophilicity (which have close relationship with the ortho phenolic dihydroxy group and the substituent at the para position of phenolic hydroxyl, respectively) make CAPE—but not CA, EF, and FA—be the major biologically active principles in propolis. The strong iron-chelating ability of CAPE in cells may also make it be a potent anti-tumor agent, which could limit tumor growth by depleting iron. This study provided a novel insight into the development of more potent iron chelators and the antioxidant mechanism of natural phenolic compounds. 

## 4. Conclusions

This study proposed that the esterification of the carboxylic moiety with phenethyl significantly enhanced the iron-binding ability and lipophilicity of CAPE, which is also responsible for its potent protection against iron-mediated cellular DNA damage. A study on the iron coordination mechanism of such natural polyphenol antioxidants will help to design more effective antioxidants for the treatment and prevention of diseases caused by metal-induced oxidative stress, as well as help to understand the structure–activity relationships of these compounds.

## Figures and Tables

**Table 1:** The LogK_ow_ values of CAPE and its analogues.

Compounds	LogKow
CAPE	2.01 ± 0.02
CA	−0.98 ± 0.02
CAME	1.27 ± 0.06
CAEE	1.51 ± 0.03
EF	1.38 ± 0.02
FA	−0.77 ± 0.01

## Data Availability

Not applicable.

## Supplementary Materials

The following are available online at https://www.mdpi.com/article/10.3390/antiox10050798/s1.
