# Supplementary material for: Caffeic Acid Phenyl Ester (CAPE) Protects against Iron-Mediated Cellular DNA Damage through Its Strong Iron-Binding Ability and High Lipophilicity
Source: Antioxidants (Basel). 2021 May 18;10(5):798. doi: 10.3390/antiox10050798 (PMC8157578; doi:10.3390/antiox10050798)
Supplement: Supplementary file 1 [file antioxidants-10-00798-s001.zip › antioxidants-1201247-SI.pdf]

Caffeic Acid Phenyl Ester (CAPE) Protects against Iron-mediated Cellular DNA Damage through its Strong Iron-Binding Ability and High Lipophilicity

Bo Shao[1,2], Li Mao[2,*], Miao Tang[2], Zhu-Ying Yan[2], Jie Shao[2], Chun-Hua Huang[2], Zhi-Guo Sheng[2], and Ben-Zhan Zhu[2,3,*]

1 Department of Public Health, Jining Medical University, Jining, Shandong, P. R. China 272067; shaobo2016@sina.com;

2 State Key Laboratory of Environmental Chemistry and Ecotoxicology, Research Center for Eco-Environmental Sciences and University of Chinese Academy of Sciences, The Chinese Academy of Sciences, Beijing, P. R. China 100085; shaobo2016@sina.com; limao@rcees.ac.cn; tangmiaosdu@163.com; yan_zhuying@126.com; jieshao@rcees.ac.cn; bjsyhch@126.com; szg326@163.com; bzhu@rcees.ac.cn;

3 Joint Institute for Environmental Science, Research Center for Eco-Environmental Sciences and Hong Kong Baptist University, Beijing/Hong Kong, P. R. China; bzhu@rcees.ac.cn;

* Correspondence: bzhu@rcees.ac.cn; limao@rcees.ac.cn; Tel.: +86-10-62849030

2. Materials and Methods

2.1 Immunofluorescence analysis of intracellular γ-H2AX generation in Hela cells

Fixed cells were permeabilized with 0.25% Triton-X100 in PBS for 10 min and blocked in 10% normal goat serum, 0.1% tween 20 in PBS for 1 hour. The cells were incubated with primary monoclonal antibody, γ-H2AX (Ser139, 20E3, Rabbit, mAb #9718) (1:600) overnight at 4°C, and then stained secondary antibodies, anti-rabbit Alexa-488 and anti-mouse (Cell Signaling Technology) for 1 hour. After Hoechst 33342 (10 μg/mL) staining for 2 min, dishes were mounted in ProLong™ Gold Antifade Mountant (Invitrogen™) and stored at 4°C in the dark before confocal laser-scanning by Broadband Confocal Leica TCS SP5. The CLSM images were obtained with the following excitation and emission wavelength: 350 nm, 460 nm for Hoechst 33342 and 488 nm, 525 nm for Alexa-488, respectively. Cells were washed 3 times with PBS (200 μL/well) at intervals.

2.2 DNA strand breakage

The experiments were conducted via incubation of plasmid pBR322 DNA (5 μg/mL) at 37 °C for 30 min in Chelex-treated HEPES buffer (100 mM, pH 7.4) with the iron-containing systems, in the absence or presence of the indicated concentrations of CAPE and its analogues.

2.3 Electron spin resonance (ESR) spin-trapping studies

ESR spectra were recorded at room temperature in a Bruker ESR 300 spectrometer. Typical spectrometer parameters were as follows: scan range, 100 G; field set, 3470 G; time constant, 200 ms; scan time, 100 s; modulation amplitude, 1.0 G; modulation frequency, 100 kHz; receiver gain, 1.25×104; and microwave power, 9.8 mW.

2.4 Oxygen consumption

The consumption of O_2 was measured by Orion 3 dissolved oxygen meter (Thermo Scientific, USA) upon mixing CAPE and its analogues with irons involving system in phosphate buffer (100 mM, pH 7.4) at 37 °C.

2.5 Fourier transform ion cyclotron resonance mass (FT-ICR-MS) detection

The mass spectrometer is equipped with 15.0T superconducting magnet and electrospray ionization (ESI) source. During the analysis, samples dissolved in methanol was directly injected into the ESI source at a flow rate of 120 μL/h. In the positive ionization mode, ultrahigh resolution mass spectrometry was obtained and broadband detection is carried out in the mass range of 150-1500 m/z. Capillary Entrance voltage was -4.0 kV, End Plate electrode voltage was -500 V. Corona Needle current was 3000 nA. The size of data points was recorded in 4 M format. 50 scans were accumulated in broad band mode for each sample.

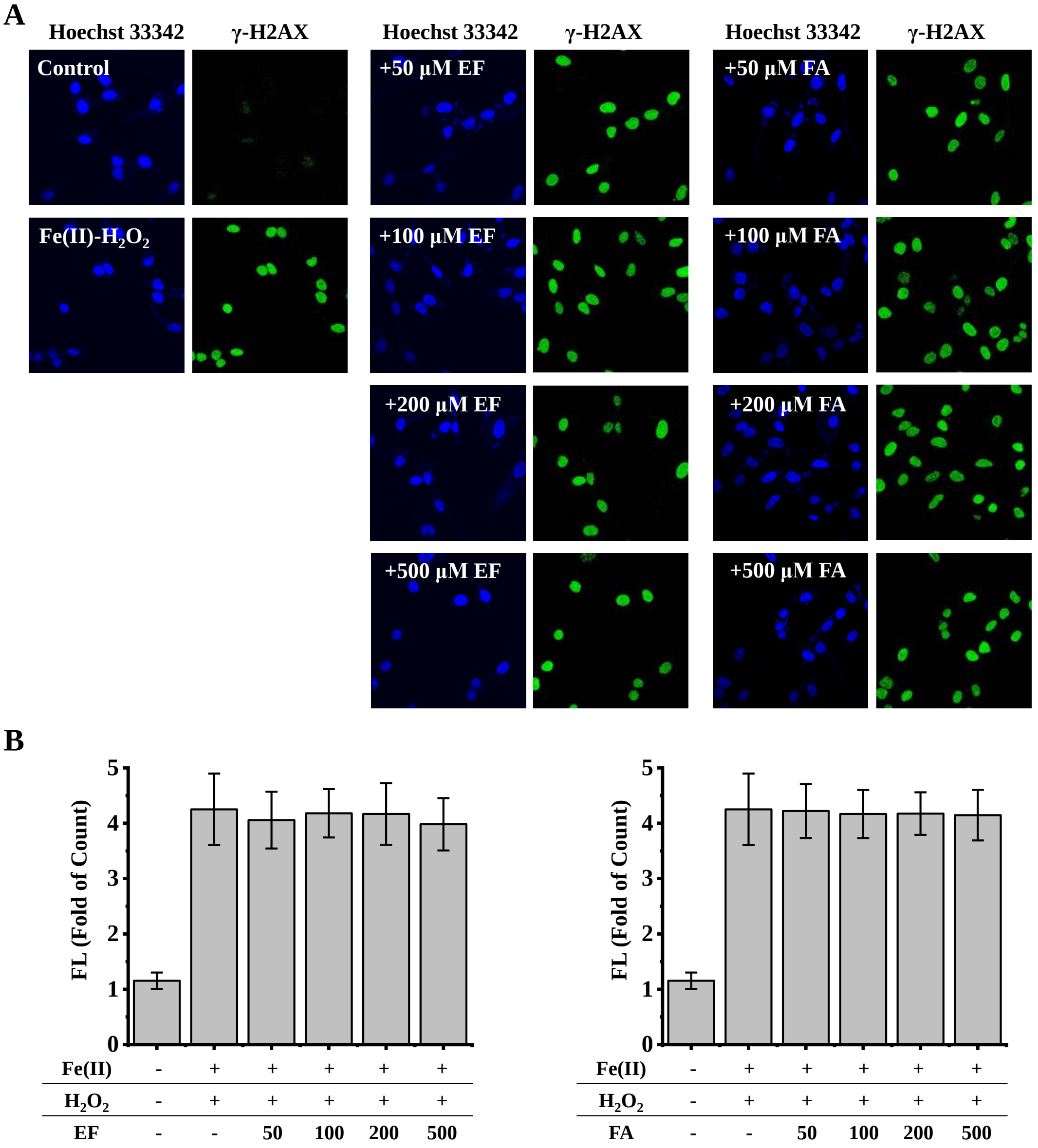

Figure. S1 Inhibition of EF and FA on the formation of γ-H2AX induced by iron-overload in Hela cells as measured by immunofluorescence staining.

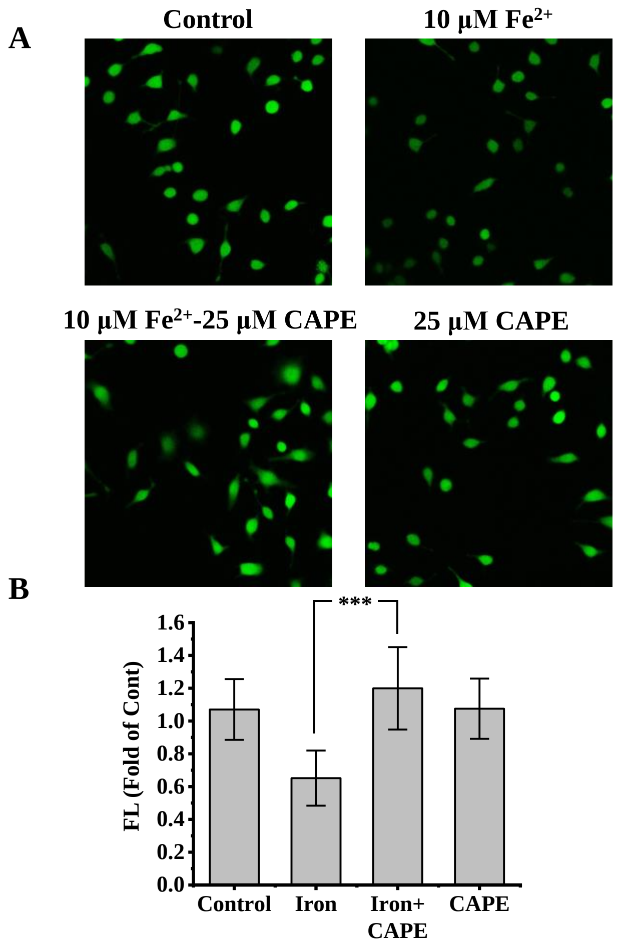

Figure. S2 Effect of CAPE on the LIP level in iron-overloaded cells as measured by CLSM. Hela cells were pretreated with 10 μM Fe(II) for 2 hours, then incubated with calcein-AM. After 15min, cells were washed and incubated with or without CAPE for 15min. The fluorescence intensity of cells was measured by CLSM. ***Significant difference from iron-overloaded group, $p < 0.001$. The experiment was repeated three times, and ten cells were randomly selected for statistical analysis in each group.

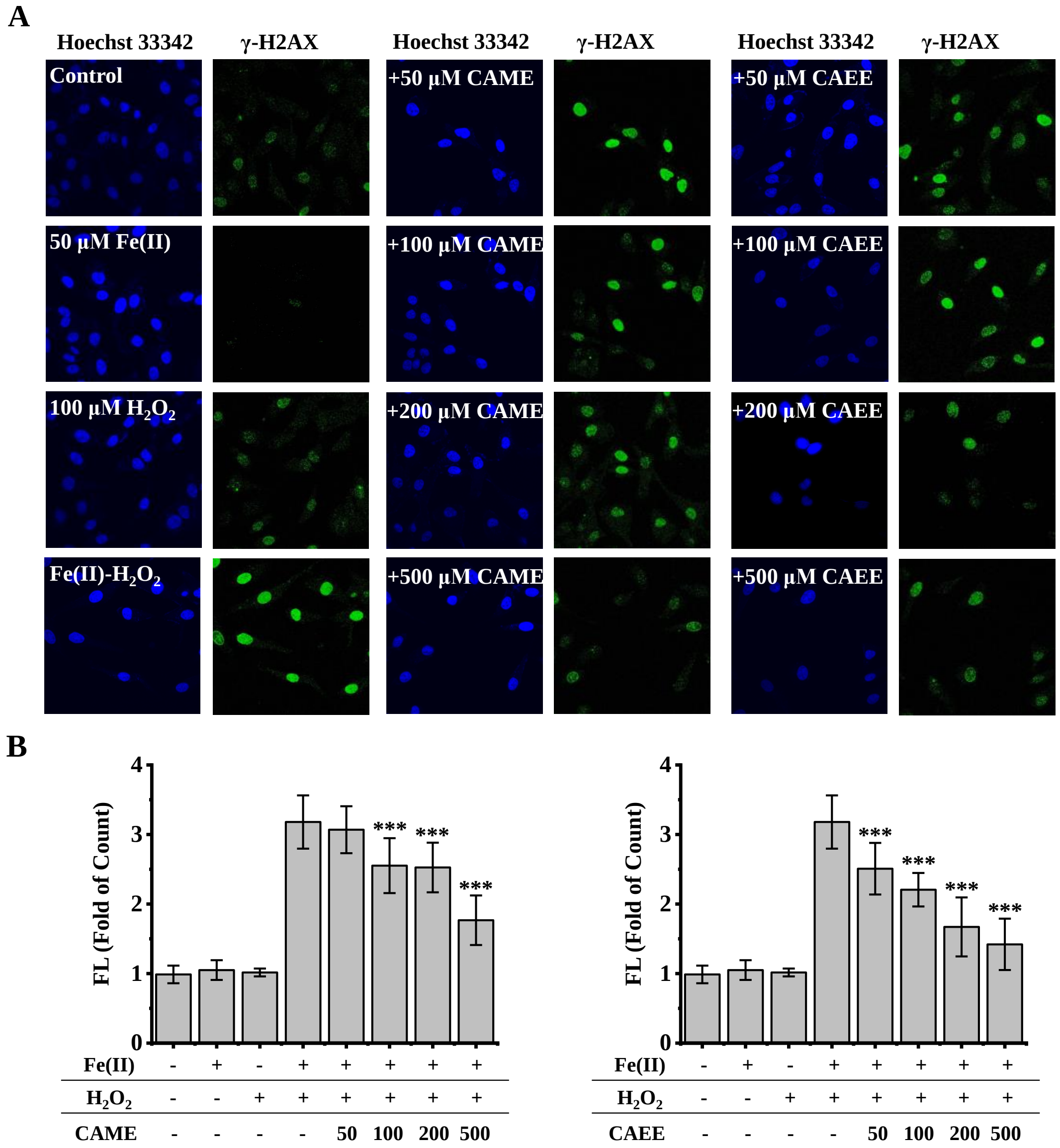

Figure. S3 Inhibition of CAME and CAEE on the formation of γ-H2AX induced by iron-overload in Hela cells as measured by immunofluorescence staining.

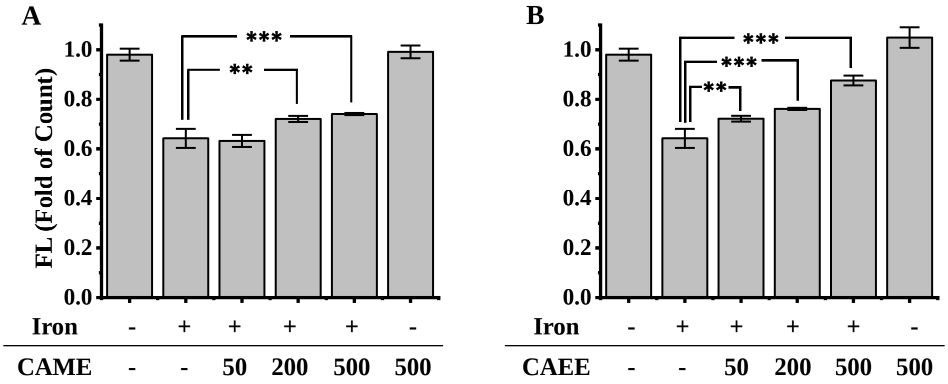

Figure. S4 Effect of CAME and CAEE on the LIP level in iron-overloaded cells as measured by flow cytometry. Hela cells were pretreated with 20 μM Fe(II) for 2 hours, then incubated with calcein-AM. After 15min, cells were washed and incubated with or without CAME or CAEE for 15min. The fluorescence intensity of cells was measured by flow cytometry. Significant difference from iron-overloaded group was marked with $^{**}p < 0.01$, $^{***}p < 0.001$. The experiment was repeated three times.

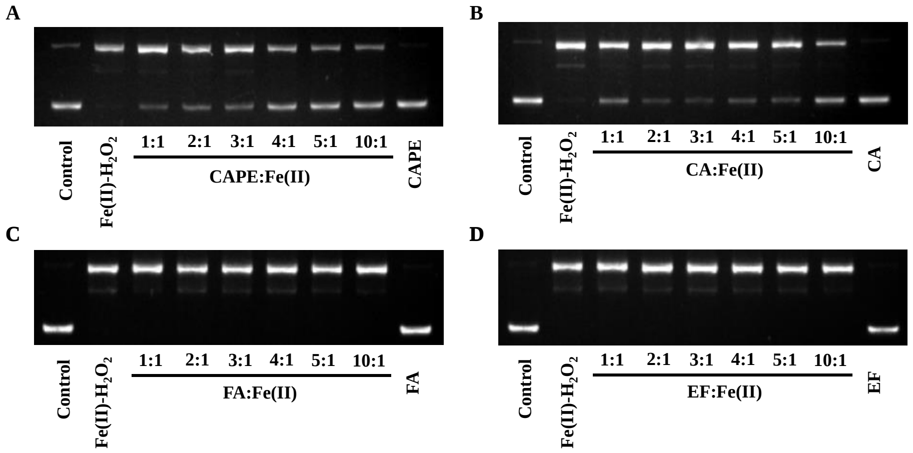

Figure. S5 Protective effect of CAPE and its analogues against Fe(II)-H2O2 induced DNA damage. The reaction mixture contained 10 μM Fe(II), 1 mM H2O2 and indicated concentrations of CAPE and its analogues.

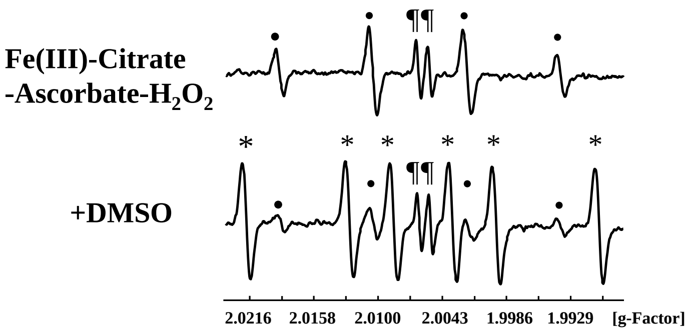

Figure. S6 ESR spectra of DMPO/•OH (•), DMPO/•CH_3 (*) and Asc•- (¶) produced by incubation of DMPO with Fe(III)-citrate, ascorbate and H_2O_2, with or without DMSO.

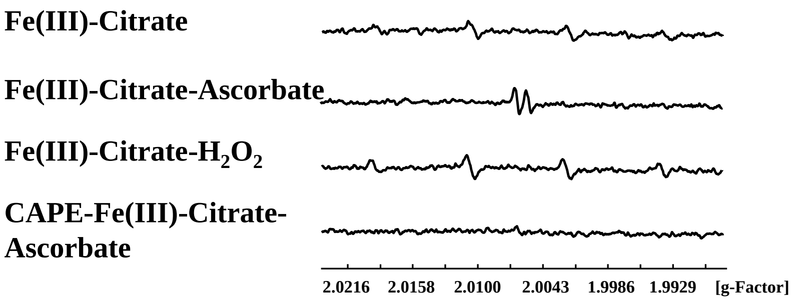

Figure. S7 ESR spectra of control group corresponding to Fe(III)-citrate-ascorbate-H_2O_2.

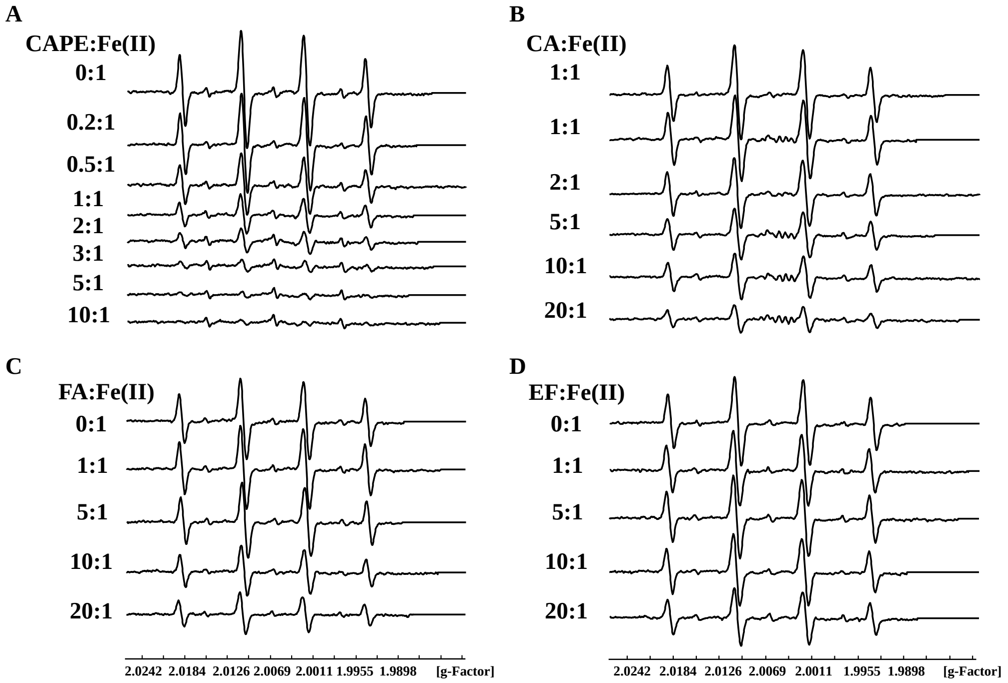

Figure. S8 Inhibitory effect of CAPE and its analogues against Fe(II)-H_2O_2 induced •OH generation. The reaction mixture contained 200 μM Fe(II) and 10 mM H_2O_2 and indicated concentrations of CAPE and its analogues.

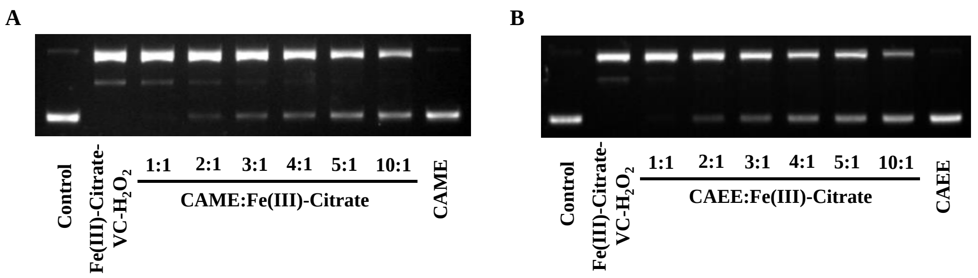

Figure. S9 Protective effect of CAME and CAEE against Fe(III)-citrate- ascorbate -H_2O_2 induced DNA damage.

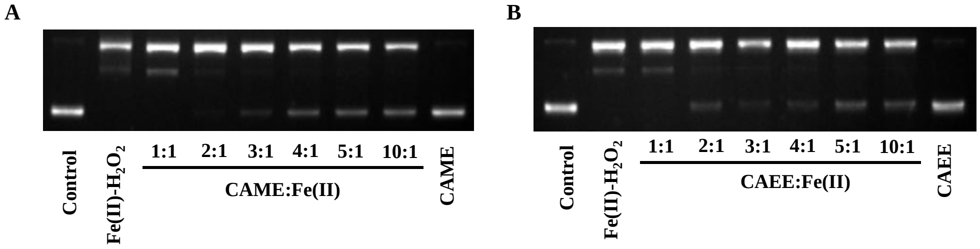

Figure. S10 Protective effect of CAME and CAEE against Fe(II)-H2O2 induced DNA damage.

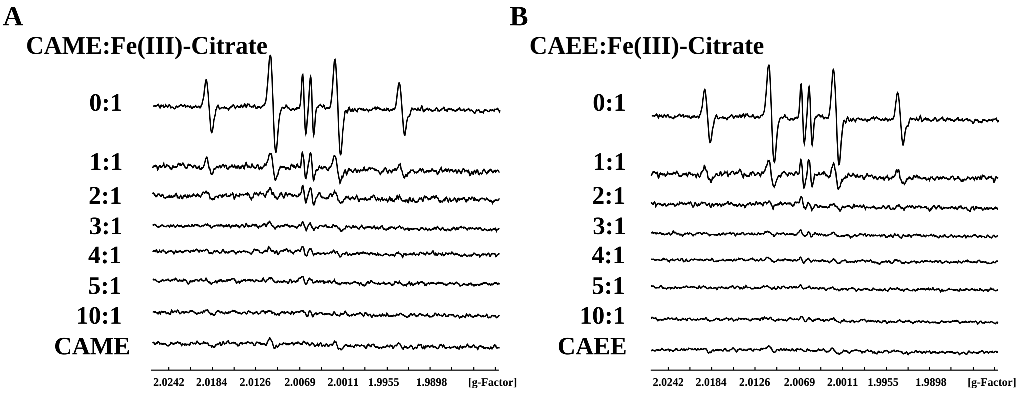

Figure. S11 Inhibitory effect of CAME and CAEE against Fe(III)-citrate-ascorbate-H_2O_2 induced •OH generation. The reaction mixture contained 100 μM Fe(III)-citrate, 100 μM ascorbate, 1 mM H2O2 and indicated concentrations of CAME and CAEE.

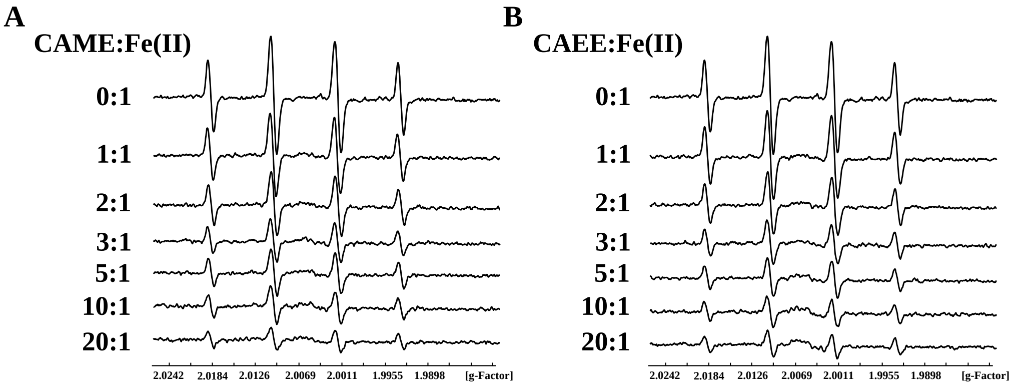

Figure. S12 Inhibitory effect of CAME and CAEE against Fe(II)-H2O2 induced •OH generation. The reaction mixture contained 200 μM Fe(II) and 10 mM H2O2 and indicated concentrations of CAPE and its analogues.

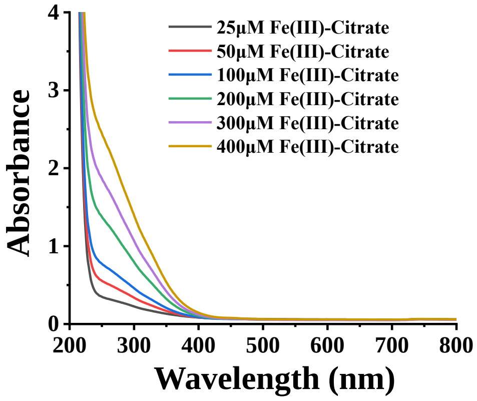

Fig. S13: UV spectral of control groups corresponding to Figure 6.

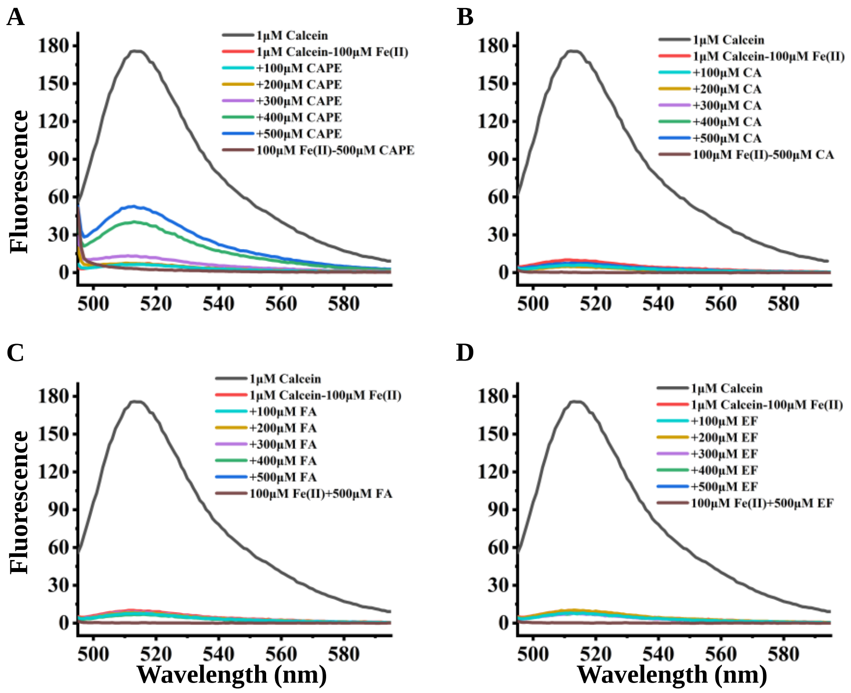

Figure. S14 Effect of CAPE and its analogues on the fluorescence intensity of calcein-Fe(II). The reaction mixture contained 1 μM calcein, 100 μM Fe(II) and indicated concentrations of CAPE or its analogues (100-500 μM). Reactions were conducted in chelex-treated HEPES buffer (20 mM, pH 7.4) at room temperature.

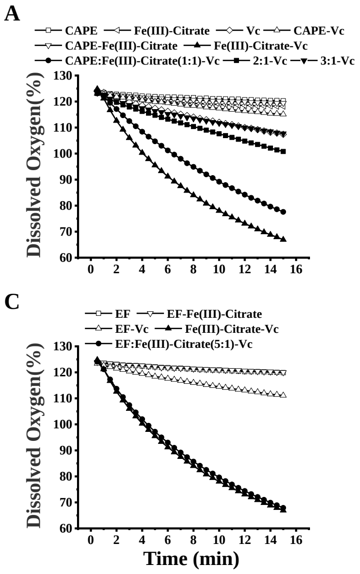

Figure S15 Inhibition of Fe(III)-citrate-ascorbate mediated O_2 consumption by CAPE and its analogues. The reaction mixture contained 100 μM Fe(III)-citrate, 1 mM ascorbate and indicated concentrations of CAPE or its analogues.

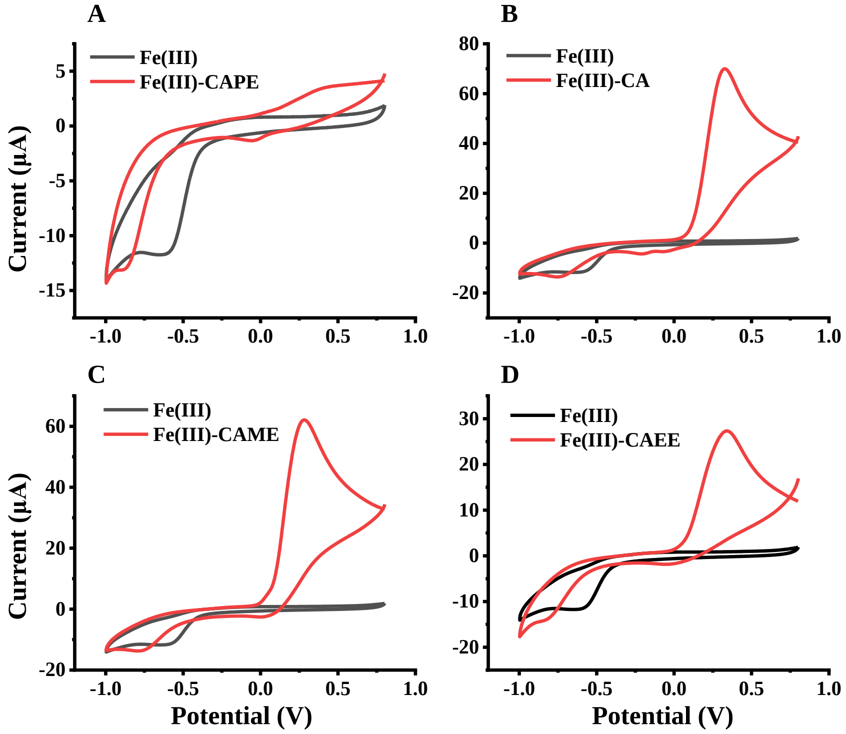

Figure. S16 Cyclic voltammograms for Fe(III) in the absence or presence of CAPE, CA CAME and CAEE.

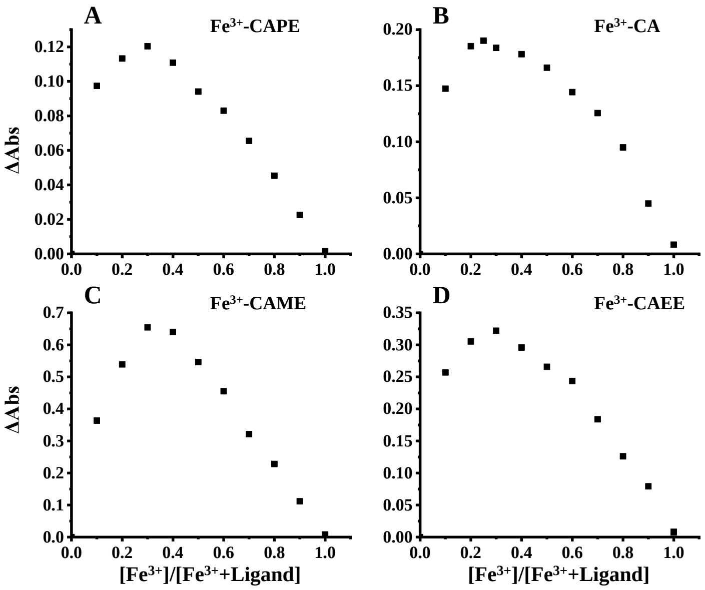

Figure. S17 Job's plot for determining the binding stoichiometry of Fe(III) and ligands including CAPE, CA, CAME and CAEE in ammonium acetate buffer (100 mM pH 6.0).

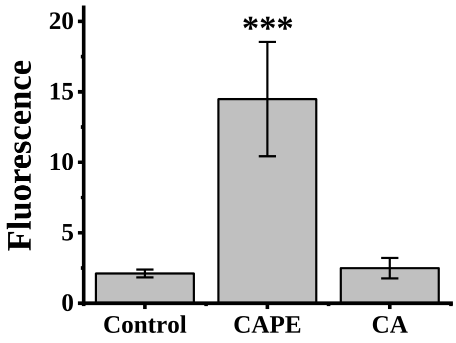

Figure. S18 Fluorescence intensity of cells after incubation with CAPE or CA. Hela cells were incubated with CAPE or CA for two hours. Subsequently, cells were washed and collected by centrifugation, the fluorescence of cells (105) was monitored by Thermo Scientific Varioskan Flash (Thermo Fisher scientific, USA) ***Significant difference from iron-overloaded group, $p < 0.001$.

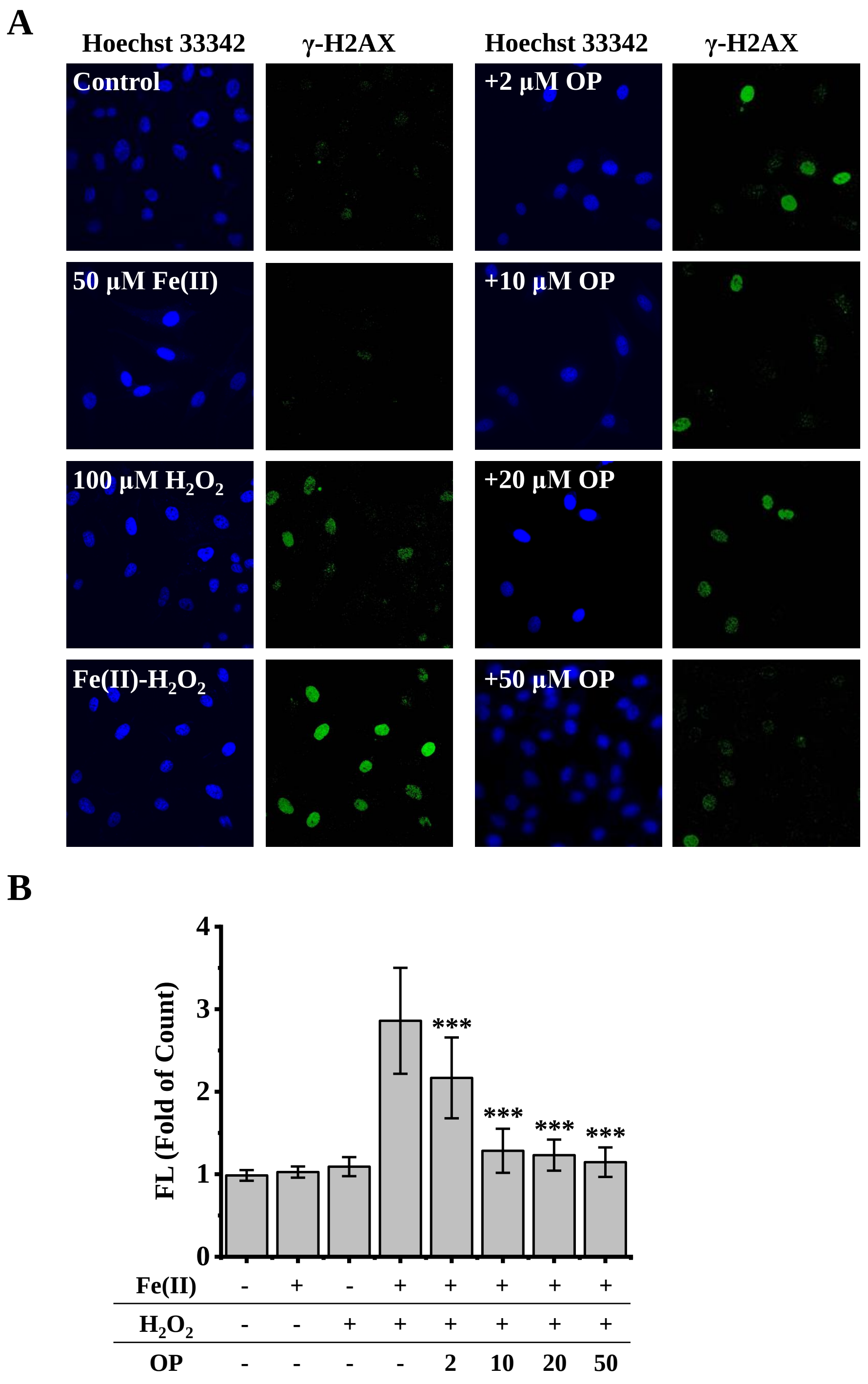

Figure. S19 Inhibition of 1,10-phenanthroline (OP) on the formation of γ-H2AX induced by iron-overload in Hela cells as measured by immunofluorescence staining.

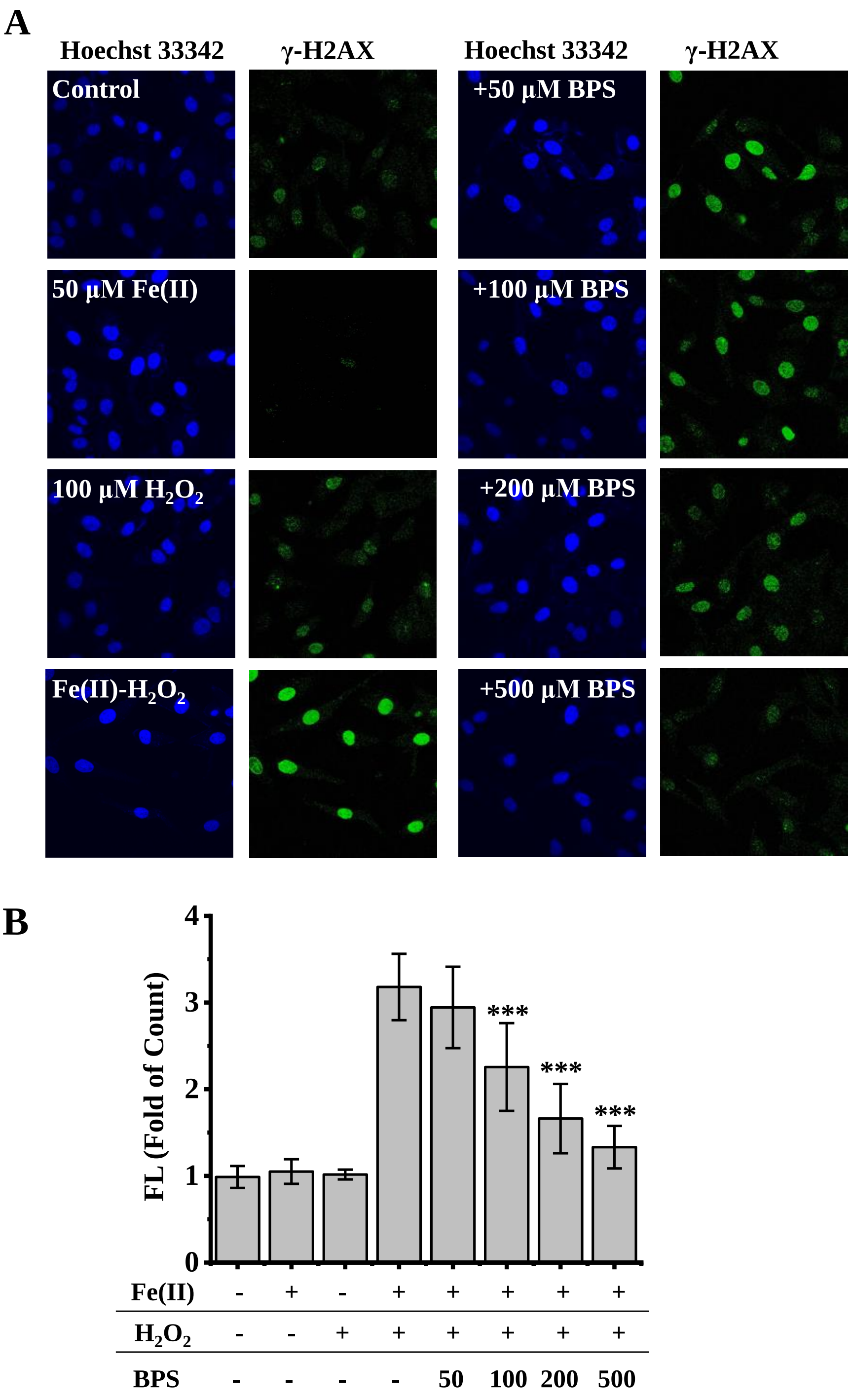

Figure. S20 Inhibition of bathophenanthroline disulphonate (BPS) on the formation of γ-H2AX induced by iron-overload in Hela cells as measured by immunofluorescence staining.

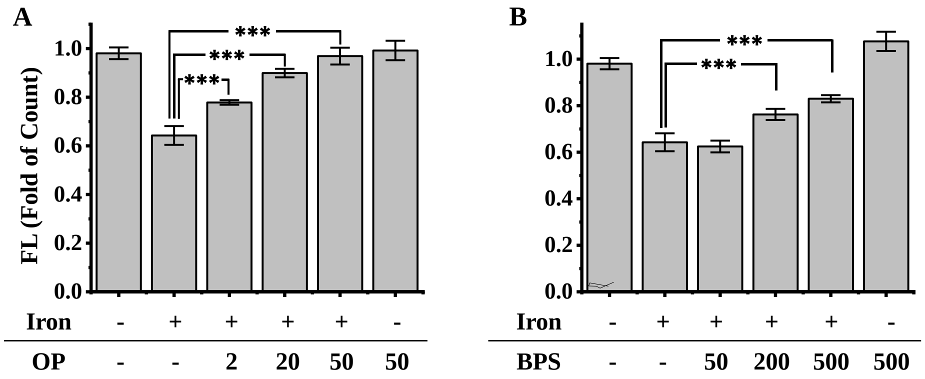

Figure. S21 Effect of OP and BPS on the LIP level in iron-overloaded cells as measured by flow cytometry. Hela cells were pretreated with 20 μM Fe(II) for 2 hours, then incubated with calcein-AM. After 15min, cells were washed and incubated with or without OP or BPS for 15min. The fluorescence intensity of cells was measured by flow cytometry.

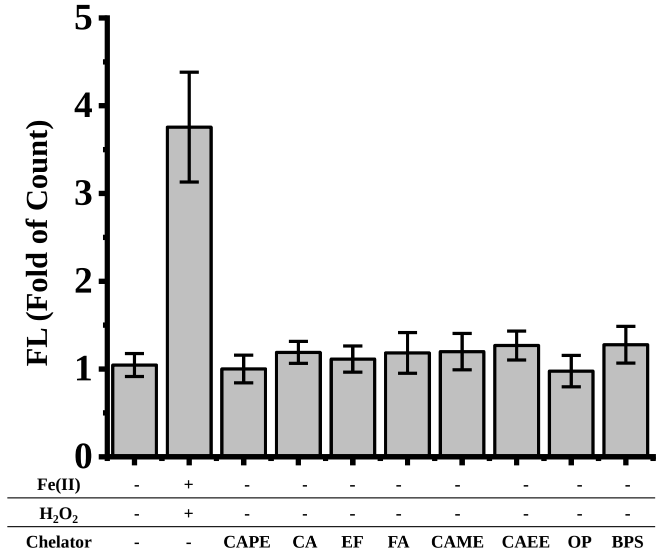

Fig. S22: Immunofluorescence staining analysis control groups.

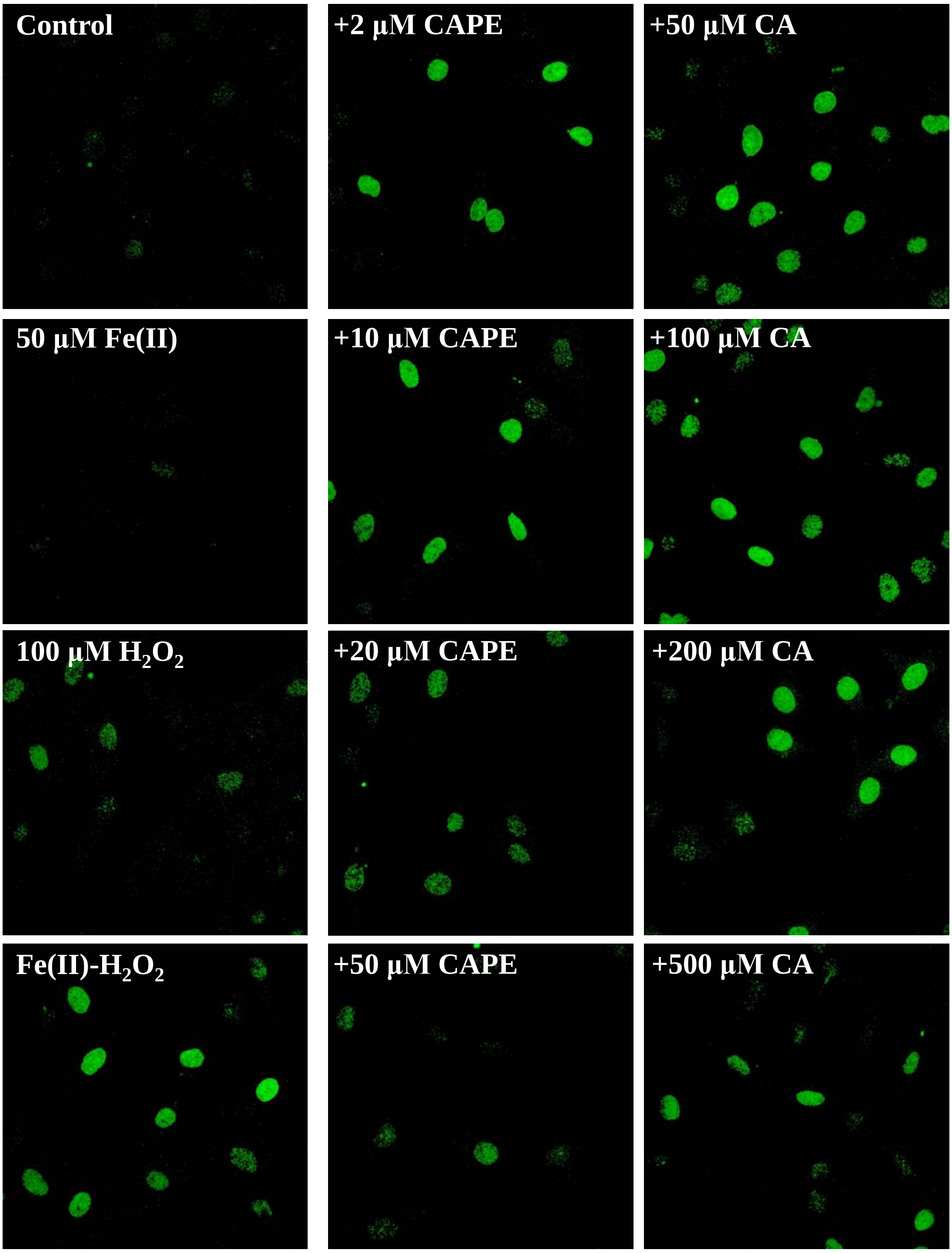

Figure S23 Inhibition of CAPE and CA on the formation of γ-H2AX induced by iron-overload in Hela cells as measured by immunofluorescence staining. (Larger picture corresponding to Figure 2)

Table S1 The binding constant between Fe(III) and ligands including CAPE, CA,CAME and CAEE.

	K_b/M-1
CAPE	$0.634*10^{22}$
CA	$<10^{20}$
CAME	$<10^{20}$
CAEE	$<10^{20}$

Table S2 Fluorescence intensity of γ-H2AX in Hela cells corresponding to Figure 2.

Group	Intensity (Fold of Control)
Control	1.00±0.06
50 μM Fe(II)	1.03±0.07
100 μM H_2O_2	1.09±0.12
Fe(II)-H_2O_2	2.86±0.64
+2 μM CAPE	3.07±0.54
+10 μM CAPE	2.71±0.32
+20 μM CAPE	1.66±0.46***
+50 μM CAPE	1.26±0.23***
+50 μM CA	3.13±0.42
+100 μM CA	3.09±0.51
+200 μM CA	2.82±0.43
+500 μM CA	1.73±0.43***

Significant difference from iron-overloaded group was marked with *p < 0.05, **p < 0.01, ***p < 0.001.

Table S3 Fluorescence intensity of calcein-AM in Hela cells corresponding to Figure 3A and B

Group	Intensity (Fold of Control)
Control	0.98±0.02
20 μM Fe(II)	0.64±0.04
+2 μM CAPE	0.65±0.00
+20 μM CAPE	0.73±0.02**
+50 μM CAPE	0.89±0.04***
50 μM CAPE	0.97±0.03
+50 μM CA	0.63±0.02
+200 μM CA	0.64±0.01
+500 μM CA	0.70±0.02*
500 μM CA	1.00±0.02

Significant difference from iron-overloaded group was marked with *p < 0.05, **p < 0.01, ***p < 0.001.

Table S4 Fluorescence intensity of calcein-AM in Hela cells corresponding to Figure 3C and D

Group	Intensity (Fold of Control)
Control	1.00±0.01
20 μM Fe(II)	0.63±0.01
+50 μM FA	0.63±0.02
+200 μM FA	0.64±0.01
+500 μM FA	0.64±0.04
500 μM FA	0.99±0.01
+50 μM EF	0.65±0.03
+200 μM EF	0.62±0.02
+500 μM EF	0.61±0.02
500 μM EF	0.99±0.01
